# Salinity tolerance in plants. Quantitative approach to ion transport starting from halophytes and stepping to genetic and protein engineering for manipulating ion fluxes

**DOI:** 10.3389/fpls.2015.00873

**Published:** 2015-10-27

**Authors:** Vadim Volkov

**Affiliations:** Faculty of Life Sciences and Computing, London Metropolitan UniversityLondon, UK

**Keywords:** salinity tolerance, halophyte, ion channel, ion transporter, ion fluxes, systems biology, synthetic biology, protein engineering

## Abstract

Ion transport is the fundamental factor determining salinity tolerance in plants. The Review starts from differences in ion transport between salt tolerant halophytes and salt-sensitive plants with an emphasis on transport of potassium and sodium via plasma membranes. The comparison provides introductory information for increasing salinity tolerance. Effects of salt stress on ion transport properties of membranes show huge opportunities for manipulating ion fluxes. Further steps require knowledge about mechanisms of ion transport and individual genes of ion transport proteins. Initially, the Review describes methods to measure ion fluxes, the independent set of techniques ensures robust and reliable basement for quantitative approach. The Review briefly summarizes current data concerning Na^+^ and K^+^ concentrations in cells, refers to primary thermodynamics of ion transport and gives special attention to individual ion channels and transporters. Simplified scheme of a plant cell with known transport systems at the plasma membrane and tonoplast helps to imagine the complexity of ion transport and allows choosing specific transporters for modulating ion transport. The complexity is enhanced by the influence of cell size and cell wall on ion transport. Special attention is given to ion transporters and to potassium and sodium transport by HKT, HAK, NHX, and SOS1 proteins. Comparison between non-selective cation channels and ion transporters reveals potential importance of ion transporters and the balance between the two pathways of ion transport. Further on the Review describes in detail several successful attempts to overexpress or knockout ion transporters for changing salinity tolerance. Future perspectives are questioned with more attention given to promising candidate ion channels and transporters for altered expression. Potential direction of increasing salinity tolerance by modifying ion channels and transporters using single point mutations is discussed and questioned. An alternative approach from synthetic biology is to create new regulation networks using novel transport proteins with desired properties for transforming agricultural crops. The approach had not been widely used earlier; it leads also to theoretical and pure scientific aspects of protein chemistry, structure-function relations of membrane proteins, systems biology and physiology of stress and ion homeostasis. Summarizing, several potential ways are aimed at required increase in salinity tolerance of plants of interest.

Salinity is among the most serious problems for modern agriculture with the estimated annual losses nowadays being over USD 12 billion (Pitman and Läuchli, 2002; Shabala, 2013). More than 20% of irrigated lands and up to 50% (Flowers, 1998) are affected by salinity; it essentially reduces the yield of agricultural crops since most of them are salt-sensitive glycophytes (Munns and Tester, 2008). The collapse of Sumer civilization about 4000 years ago was caused by improper agricultural techniques, which led to soil salinization and drop in the agricultural productivity in the area (Serrano, 1996; Pitman and Läuchli, 2002). However, progress in modern agriculture and science rather allowed to set problems and pose questions than to get clear answers. We still do not know how to deal with salinity and to grow plants under salinization.

Effects of salinity on living plants include osmotic stress, toxic effects of high salt, mostly sodium ions, and corresponding oxidative stress (Flowers, 2004; Munns and Tester, 2008; Shabala, 2013). Osmotic stress is proportional to concentration of external salt solution, usually with osmotic pressures over 1 MPa. Toxic effect of sodium ions results from their rise in cytoplasm of plant cells. Developing in plant cells oxidative stress adds to the negative effects of salinity on the whole plants.

Ion transport via cell membranes is the basic factor determining salinity tolerance. Ion fluxes control ion concentrations; finally the values and regulation of the fluxes are essential for salinity tolerance. The review starts from brief description of ion transport in halophytes. Then it provides basic details and features of ion transport aiming to understand what could be altered and the potential results of the changes. Further discussion is directed to specific transporters and to genetically modified and artificially designed transport proteins for modulating ion transport under conditions of salinization.

## Ion Transport in Halophytes and Effects of Salt Stress on Membrane Transport in Glycophytes

**FIGURE 1:**
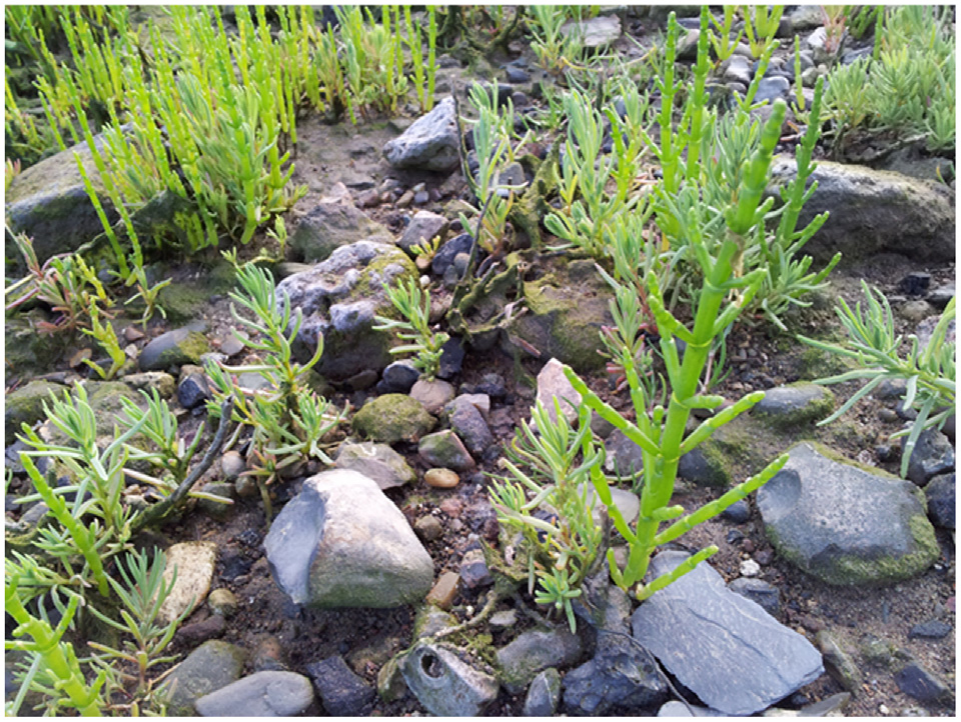
**Halophytes *Salicornia sp.* (larger and bright green plants at the picture) and *Suaeda maritima* (smaller and grayish-green plants at the picture) are growing at the salt-affected soil near river Medway in the UK, where the area is flooded with mixed sea and river water under high tides (Beginning of June 2014).** The size of the largest specimen is about 15 cm.

Brilliant examples of salinity tolerance are represented by halophytes, which are able to grow at high concentrations of salt (**Figure 1**), under irrigation by seawater and even under several times higher salt concentrations than in seawater (over 2 M of NaCl) (Flowers et al., 1977; Flowers and Colmer, 2008). Halophytes are usually considered like sodium tolerant plants. Indeed, NaCl is the main source of salinity in most areas, though chloride, sulfate, calcium, magnesium, and the other ions are involved constituting sometimes the main salt for soil and water salinization (Flowers et al., 1977). “Domestication” of halophytes is one more way to use salt-affected and salinized territories.

Halophytes is an undisputable example and proof that salinity tolerance in plants is achievable. The main questions are:

– what are the salinity tolerance traits in halophytes;
– how to bring the relevant trait or multiple traits to agricultural plants;
– to which extent the transfer could be realized without essentially influencing the growth rate, agricultural productivity and quality of grain, fruits, edible parts, flowers or the other economically important elements/features/traits of agricultural plants.

The simplest idea under salinity is to decrease sodium net influx and increase potassium net uptake via plasma membrane of epidermal root cells to alter mineral nutrition. Surprisingly, in most cases the pure straightforward and strict approach is not realized neither in halophytes, nor in glycophytes (Flowers, 2004; Maathuis et al., 2014). Halophytes imply several strategies to cope with high concentrations of salts including (1) sodium exclusion from roots, (2) accumulation of high sodium in shoots, (3) shedding specialized leaves, (4) localizing salts in vacuoles, (5) excreting salts via salt glands etc. The role and contribution of each strategy depend on the habitat and type of a halophyte plant (Flowers et al., 1977; Breckle, 2002; Lüttge, 2002; Flowers and Colmer, 2008; Munns and Tester, 2008; Shabala, 2013). So far the known transport systems in halophytes are basically the same like in glycophytes due to common ancestry and evolution (Flowers et al., 2010): the trait of salt tolerance emerged independently over 70 times in different groups of grasses (Bennett et al., 2013). The knowledge facilitates potential transfer of salinity tolerance traits to agriculturally important plants.

**FIGURE 2:**
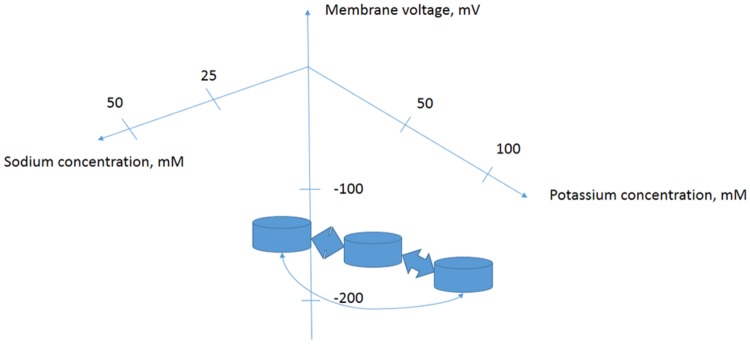
**Proposed simplified model of several attractors for a plant cell with specific metabolic and regulatory networks determined by cytoplasmic ion concentrations and membrane potential.** Changes in extracellular ion concentrations result in transition of cells from one state of concentrations-membrane potential-protein and DNA–RNA expression levels and activity pattern to another one. Stability of the physiological states and trajectories of transitions could be studied in more detail using complex approaches of ionomics–metabolomics–proteomics–nucleic acids expression arrays and biophysical methods.

It is important to consider living cell as a complex system. There are networks of signaling events and metabolic reactions. Under salinity the increase in cytoplasmic Na^+^ and reduction of K^+^ result in changes of membrane potential, osmotic pressure, turgor pressure, calcium signaling, reactive oxygen species signaling, transcriptional regulation, alteration of gene expression, modification of protein expression pattern and spectra of siRNAs, signaling molecules, phytohormones and metabolites. The set of parameters including ion fluxes characterizes cell in a definite physiological state. Transition from one physiological state to the other one could be described by a trajectory in a multidimensional space, while the stable physiological states are considered as dynamic attractors. The volume and shape of the attractor in multidimensional space could be registered using means of “omics” that is RNA expression microarrays, proteomics, ionomics, metabolomics etc. (**Figure 2**). Properties of the attractors are slowly studied and understood for biological systems (Spiller et al., 2010; Breeze et al., 2011), though the idea is initially well developed in physics, especially in plasma physics for non-equilibrium thermodynamic systems (Akhromeeva et al., 1989, 1992). Obviously and intuitively the biological systems with tens of thousands of participating expressed genes are more complex than physical systems. Salt treatment up- and down-regulates tens and hundreds of genes including transcription factors, genes of transporters and regulatory proteins (e.g., Taji et al., 2004; Volkov et al., 2004; Liu et al., 2013; Takahashi et al., 2015) when the cells are moving to a new distinct physiological state of gene expression, metabolic control and ion transport.

**FIGURE 3:**
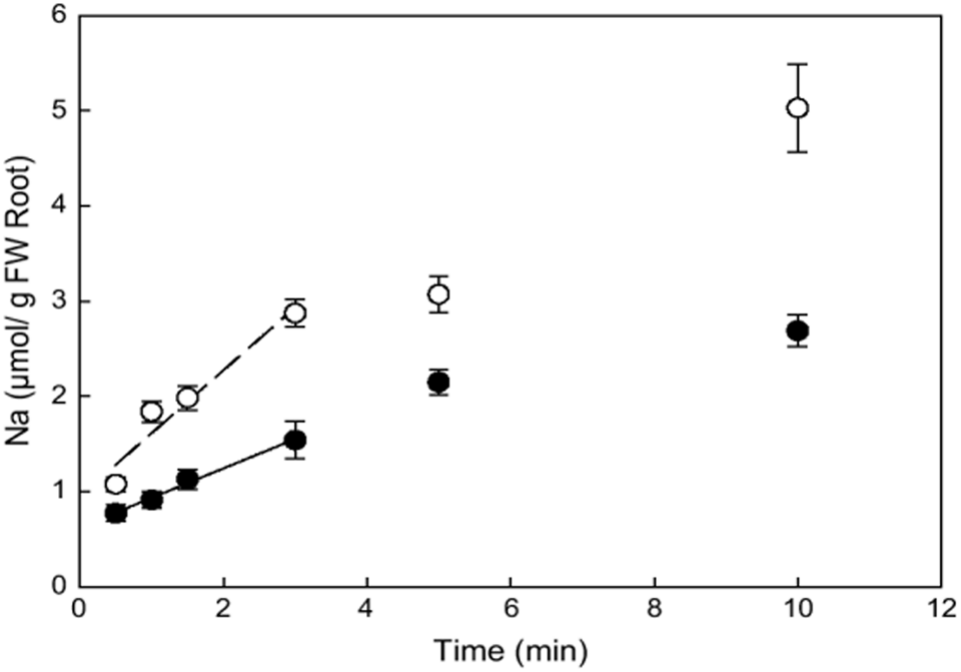
**Kinetics of initial unidirectional Na^+^ influx into roots of *Arabidopsis thaliana* (open circles) and *Thellungiella halophila* (closed circles) as determined from ^22^Na^+^ accumulation of individual plants from ^22^Na^+^ labeled nutrient solution with 100 mM NaCl and 0.1 mM CaCl_2_.** Error bars are SE (*n* = 4). Reproduced from Wang et al. (2006) with the permission from the publisher Oxford University Press.

**FIGURE 4:**
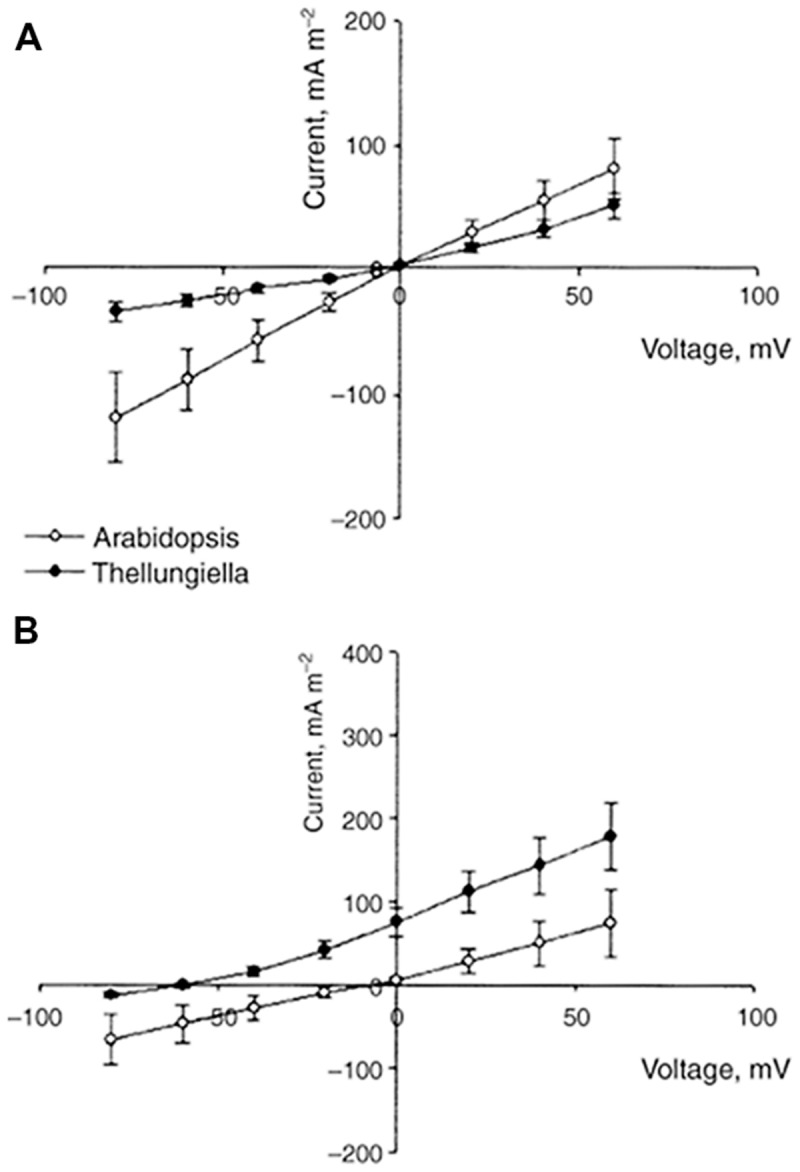
**Comparison of current–voltage curves for whole-cell instantaneous currents in root protoplasts of *A. thaliana* (open symbols) and *T. halophila* (closed symbols).** Currents are normalized to protoplast surface. The pipette solution was always 100 mM KCl. The bath solution was 100 KCl **(A)** or 100 NaCl **(B)**. Data are given as means ± SE (*n* = 6 for *A. thaliana, n* = 13 for *T. halophila*). Reproduced from Volkov and Amtmann (2006) with the permission from the publisher John Wiley and Sons. Note that IV curve for instantaneous current in *T. halophila* resembles (though not completely obeys) the expected one from Goldmann–Hodkin–Katz equation and the shift in reversal potential of ion current in 100 mM KCl/100 mM NaCl is reflected in slope of IV curve for voltages above and below the reversal potential. The electric current characterizes the ion transport properties of plasma membranes and their selectivity for K^+^ over Na^+^.

Comparison of ion fluxes via membranes between halophytes and glycophytes often demonstrates lower sodium uptake for halophytes (reviewed in: Flowers and Colmer, 2008). However, an evident problem in comparison is the high variability in ion transport between plant species because of growth rate, tissue-specific variability and the other physiological factors. It is reasonable to consider similar plants and achieve comparable values. Recent results on ion fluxes in glycophyte *Arabidopsis thaliana* and similar from the point of genome and morphology halophyte *Thellungiella halophila* demonstrated lower Na^+^ fluxes and higher K^+^/Na^+^ selectivity of ion currents in the roots and root protoplasts of the halophyte under salt treatment (**Figures 3** and **4**, and in: Volkov et al., 2004; Volkov and Amtmann, 2006; Wang, 2006; Wang et al., 2006; Amtmann, 2009; resembling results for roots of the two plants: Alemán et al., 2009). Lower Na^+^ accumulation and higher K^+^/Na^+^ ratio under salt treatment were also found in roots of leguminous halophyte *Melilotus indicus* compared to similar herbaceous glycophyte *Medicago intretexta* (Zahran et al., 2007).

Other strategies may be involved depending on the level of salinity tolerance, plant morphology, habitat and the other environmental factors and evolutionary history. For example, salt tolerant *Plantago maritima* had similar sodium uptake rates by roots compared to salt-sensitive *P. media* (Erdei and Kuiper, 1979; de Boer, 1985); the salt-tolerance in the pair is rather associated with xylem transport and sodium accumulation in vacuoles of leaf cells.

Vacuolar membranes of several halophytes were also a subject of special investigation. Electrophysiological patch clamp study of vacuoles from leaves of *Suaeda maritima* did not find any unusual features to support the high salt tolerance of the halophyte (Maathuis et al., 1992). Patch clamp experiments to compare vacuoles from roots of *P. maritima* and *P. media* also did not reveal striking differences apart from an extra smaller ion channel conductance in the tonoplast of the halophyte; salt stress essentially reduced the open probability of larger non-selective between K^+^ and Na^+^ ion channel conductance in both species but did not change the properties of the conductance (Maathuis and Prins, 1990). However, comparison of tonoplast from suspension culture cells of halophytic sugar beet with glycophytic tomato revealed rectification properties of ion channels in vacuolar membrane of the halophyte (Pantoja et al., 1989). At positive voltages in outside-out configuration (corresponding to ion currents out of vacuole) the ion-channel-like conductance dropped 6.5 times presumably preventing the transport of ions from vacuole to cytoplasm; the conductance was not selective for K^+^ over Na^+^ in both species (Pantoja et al., 1989).

**FIGURE 5:**
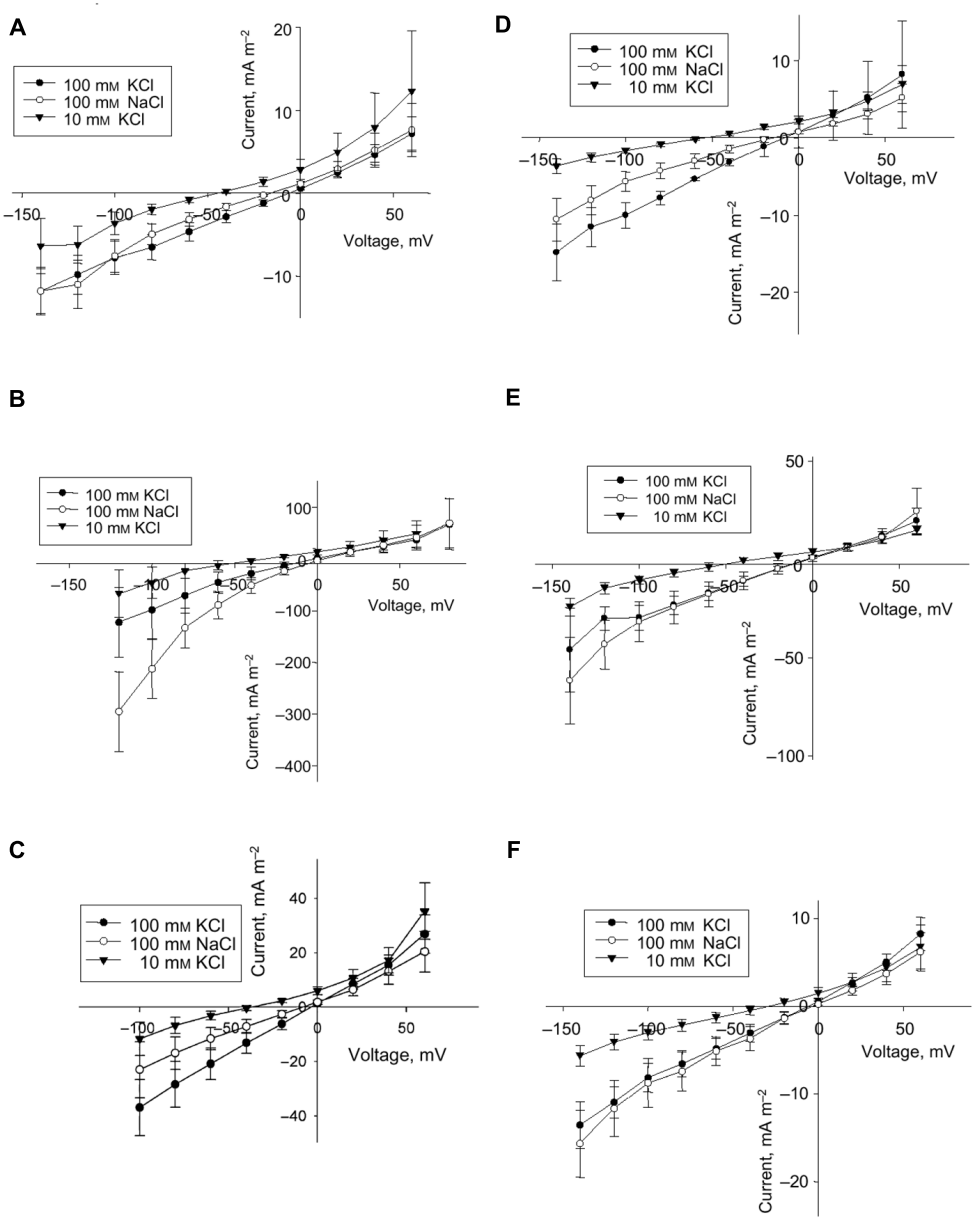
**Effect of salt stress on instantaneous current in protoplasts from the elongation zone and emerged blade portion of the developing leaf 3 of barley *(Hordeum vulgare* L.) **(A–F)**.** Averaged current–voltage (I–V) relationship in protoplasts from mesophyll **(A,D)** and epidermis **(B,E)** of the emerged blade, and from protoplasts of the elongation zone **(C,F)**. Control **(A–C)** and salt treatment **(D–F)**, *n* = 5–8 for control protoplasts and *n* = 3–6 for protoplasts for salt treatment; error bars are standard errors. Concentrations of KCl and NaCl in the bath are indicated. The pipette solution was always 100 mM KCl. Plants had been exposed to 100 mM NaCl for 3 days prior to protoplast isolation. Reproduced from Volkov et al. (2009), composed from Figures 1, 2, 4 and 5 with the permission from the publisher John Wiley and Sons.

Complexity of ion transport within the whole plant under salinity is confirmed also in experiments with glycophytes. Ion transport could be essentially influenced by salt stress in a cell-specific manner. The information is required to consider the whole plant responses to salinity and to alter them in a desired direction. An example of changes in membrane electric conductance after salt stress in several types of cells from barley leaves is given in **Figure 5**.

## Basic Assumptions for Regulating Ion Transport in Plant Cells

Before pondering ways to modulate ion transport it is helpful to describe techniques to measure ion fluxes and then introduce basic important parameters, which could be influenced and manipulated to alter ion fluxes and intracellular ion concentrations. The knowledge is also required to explain, understand and predict the results with overexpression/knockout of individual transporters and ion pumps (see below).

### Comparison between Different Methods for Measuring Ion Fluxes

Total activity of proton pumps, ion channels, transporters and potential “leak” pathways of ion transport (see below) is reflected in changing and regulated ion fluxes. The coinciding values of ion fluxes measured by several independent techniques are needed to provide reliable and robust basis for the further conclusions. Direct and indirect methods include (1) estimates based on kinetic measurements of ion concentrations (both in plants and in nutrient solution), (2) electrophysiological methods, (3) technique of measuring ion fluxes using vibrating ion-selective electrodes [microelectrode ion flux estimate/measurements (MIFE)], (4) measurements of unidirectional ion fluxes (e.g., radioactive isotope ^22^Na^+^ for sodium, while Rb^+^ is often used to imitate K^+^ fluxes) and (5) several other methods including ion-selective fluorescent and non-fluorescent indicators, potentially NMR spectroscopy, ion-conductance scanning microscopy etc. (**Table 1**).

**Table 1:** Comparative summary for methods to measure ion fluxes in plants and their tissues and cells.

Method or group of methods	Principle of the method	Spatial and temporal resolution, advantages	Disadvantages
Kinetic measurements of ion concentrations	Ion concentrations in plant tissues or in nutrient solution are measured in time, the concentration differences along the time points are plotted against time.	Resolution is at the level of whole plant or plant tissues, usually tens of minutes and hours are needed to register changes. Simple methods without a need of special equipment, convenient for most ions.	Measures net fluxes, not separate influx and flux outside of plant tissues. Low level of resolution.
MIFE: microelectrode ion flux estimate/measurements	Tiny ion-selective microelectrode with tip around a μmeter vibrates within seconds in the vicinity of a cell or plant tissue and measures ion concentrations. Changing in time difference in concentrations is recalculated to ion fluxes.	Resolution at the level of individual cells within seconds, measurements may last for hours.	Requires special equipment. Ion-selective electrodes often interfere with organic compounds. Reliable ion-selective resins available for a few major ions only.
Measurements of unidirectional ion fluxes	Plant tissues or organs are loaded with radioactive ions or rare ions to imitate ions of interest. Unidirectional usually outward ion fluxes of the isotope or rare ion are measured then as changes in concentrations against time.	Spatial resolution at the level of whole plant or plant tissues, recordings from minutes lasting to hours are needed to register changes.	Requires radioactive isotopes and often complicated calculations with several proposed pools of ions.
Electrophysiological methods	Isolated cell membrane, piece of membrane or single cell within a plant preparation are subjected to set of physiological voltages and ion current is registered in the form of electric current.	Spatial resolution of single molecules or single cells. Temporal resolution from μseconds to minutes. High accuracy and possibility to find out specific molecules for transport of definite ions.	Indirect measurements, measure total electric current carried out by several ion species.
Fluorescent indicators	Fluorescent ion selective indicators	Resolution at the level of individual cells within tens of seconds to minutes, recordings over tens of minutes to hours.	Require specific protocols for loading, intrinsic autofluorescence and non-specific adsorption of fluorescent indicators could be drawbacks.

*The distinction between the methods is partially arbitrary.*

Estimates from ion concentrations are the sum of influx and eﬄux of ions from plant cells. The measurements finally provide the important physiological information about averaged net changes and fluxes. However, the existing concentrations are often already high, therefore it takes at least hours and sometimes days to determine kinetic changes of ion concentrations and obtain kinetic curves, usually without detailed peculiarities and high temporal resolution of minutes. For example, after 25 h of 100 mM NaCl treatment sodium contents in roots of glycophyte *A. thaliana* rose from 0.2 to 2% of dry weight (DW) of the roots (0.087–0.87 mmole Na^+^ per g of DW), while in halophyte *T. halophila* sodium in roots increased from 0.15 to 1.2% (0.065–0.52 mmole Na^+^ per g of DW) (Volkov et al., 2004). Fast initial rates of net Na^+^ uptake by roots during the first 6 h were 0.064 μmole/(g root DW^∗^min) in *Thellungiella* and 0.048 μmole/(g root DW^∗^min) in *Arabidopsis*. After 24 h of salt treatment, the rates dropped to 0.0004 nmole/(g root DW^∗^min) in *Thellungiella* and 0.003 nmole/(g root DW^∗^min) in *Arabidopsis* (Wang, 2006).

To elucidate detailed mechanisms of ion transport it is essential to add the other methods. One more requirement is to compare ion fluxes measured by different techniques: changes of ion concentrations are expressed in moles/g of fresh or dry weight (FW or DW), while electrophysiological and MIFE measurements are normalized per a unit of surface area, A/m^2^ and moles/(m^2∗^s), correspondingly. Rough estimate for conversion is that DW is about 10–15% of FW; for recalculation per surface area the size of roots or protoplasts is required. Estimates relating potassium flux in epidermal cells to ion electric currents were done for rye roots (White and Lemtiri-Chlieh, 1995). The net K^+^ flux was estimated 1.0–1.9 μmole/(g root WF^∗^h) and unidirectional K^+^ flux was about 7 μmole/(g root WF^∗^h) from mineral solution with 0.6 mM K^+^ (White et al., 1991). Epidermal cells with diameter 26 μm were considered 8.3% of root volume; then the net fluxes in μmole/(g root WF^∗^h) corresponded to 0.11–0.21 pmole/(cell^∗^hour) or 3.1–5.9 pA/cell. Unidirectional flux of 7 μmole/(g root WF^∗^h) corresponded to 0.77 pmole/(cell^∗^hour) or 21.7 pA/cell (White and Lemtiri-Chlieh, 1995). Converting per surface area of the whole protoplasts, the fluxes of 0.11–0.21 pmole/(cell^∗^hour) correspond to 14–27 nmole/(m^2∗^s) and 0.77 pmole/(cell^∗^hour) ≈ 100 nmole/(m^2∗^s), the values reasonably confirmed by the other methods (see below). Conversion of the flux in pmoles to number of ions per a cell gives 0.77 pmole/(cell^∗^hour) ≈ 1.3 ^∗^ 10^8^ ions/(cell^∗^second) (see below).

Electrophysiological techniques of two-electrode voltage clamp or patch clamp measure ion currents across membrane of plant cells (protoplasts for patch clamp) under determined applied voltage via the membrane and provide current–voltage curves with resolution up to pA and μseconds (**Figures 4** and **5**). For the example of patch clamp study with root protoplasts of *Arabidopsis* and *Thellungiella*, the inward K^+^ fluxes in external 100 mM KCl at -80 mV were 120 mA/m^2^ = 120 fA/μm^2^ ≈ 1.2 10^-18^ mole/(μm^2∗^s) = 1.2 μmole/(m^2∗^s) for *Arabidopsis* and 30 mA/m^2^ ≈ 300 nmole/(m^2∗^s) for *Thellungiella*, correspondingly. Inward Na^+^ fluxes under the same conditions, but in 100 mM NaCl in the external medium are 70 mA/m^2^ ≈ 700 nmole/(m^2∗^s) for *Arabidopsis* and 15 mA/m^2^ ≈ 150 nmole/(m^2∗^s) for *Thellungiella* (Volkov and Amtmann, 2006).

Comparisons of electric currents and ion fluxes were performed in a series of simultaneous measurements using patch clamp and MIFE for wheat root protoplasts, when MIFE measured H^+^ fluxes (Tyerman et al., 2001). Proton fluxes in the experiments basically correlated to electric currents, though with large variation between protoplasts (Tyerman et al., 2001). Further on MIFE proved the lack of “dark” electroneutral fluxes for K^+^ transport in the wheat root protoplasts (the situation was different for Ca^2+^, large Ca^2+^ fluxes were not electrogenic): K^+^ ion currents via outward and inward potassium channels nearly exactly corresponded to the K^+^ ion fluxes measured by ion selective electrodes of MIFE (Tyerman et al., 2001; Gilliham et al., 2006). Some deviations from 1:1 ratio and variation between protoplasts were explained by uneven distribution of ion channels (Gilliham et al., 2006). Potentially atomic force microscopy or ion-conductance scanning microscope with further ion channel recordings (e.g., Hansma et al., 1989; Korchev et al., 1997; Lab et al., 2013 for ion-conductance scanning microscope) could be useful to explore the distribution of lipid rafts and clusters of ion channels at nanometre resolution.

Values of ion fluxes measured by patch clamp in protoplasts are usually higher than fluxes measured by MIFE for intact roots (e.g., Shabala and Lew, 2002; Chen et al., 2013), but coincide within orders of magnitude and essentially depend on composition of ambient medium, concentration of ions, membrane potential of cells and on multiple physiological factors (e.g., Ivashikina et al., 2001; Shabala et al., 2009). Experiments with MIFE are non-invasive and simpler; therefore provide huge opportunities with temporal resolution of seconds and spatial resolution within less than tens of microns for exploring physiological factors and conditions, which influence ion fluxes to and out of cells (Newman et al., 1987; Newman, 2001; Shabala, 2006; Sun et al., 2009; Shabala and Bose, 2012). Ion fluxes of K^+^, Na^+^, and other ions in the vicinity of roots were measured by MIFE and compared for different salt-tolerant and salt-sensitive cultivars and agricultural species (Chen et al., 2005; Cuin et al., 2008, 2012), along root zones of several plants (Garnett et al., 2001; Chen et al., 2005; Pang et al., 2006), for mutants in specific ion channels or transporters (e.g., Shabala et al., 2005; Demidchik et al., 2010), under treatment by physiologically active compounds (Cuin and Shabala, 2007a; Shabala et al., 2009; Pandolfi et al., 2010; Demidchik et al., 2011; Ordoñez et al., 2014), after generation of reactive oxygen species and salt stress (Cuin and Shabala, 2007b; Demidchik et al., 2010) and for numerous other conditions. MIFE is also a good method for studying ion transport in cell biology (Lew et al., 2006; Valencia-Cruz et al., 2009; Demidchik et al., 2010). The results provided huge volume of information about characteristics, kinetics and physiological features of ion fluxes under salt stress, helped to develop fast tests for salinity tolerance (Chen et al., 2005; Cuin et al., 2008). The results are described in hundreds of publications and several reviews (e.g., Newman, 2001; Shabala, 2006; Sun et al., 2009; Shabala and Bose, 2012), so are not covered here in more detail. Among the limitations of MIFE is the selectivity of ion-selective electrodes, which is influenced by interfering ions (e.g., Knowles and Shabala, 2004) and sometimes affected by physiologically active compounds and proteins in the medium for measurements after interaction with the material of ion-selective electrodes (e.g., Chen et al., 2005), hence demanding more control checks.

Unidirectional fluxes of ^22^Na^+^ for sodium and ^42^K^+^ or Rb^+^ (a tracer for K^+^) provide fast kinetics of ion transport within tens of seconds as determined by the speed of sampling and changes in concentrations at the background of initial level without ^22^Na^+^ and ^42^K^+^/Rb^+^ (e.g., **Figure 5** for ^22^Na^+^ fluxes). The method is used widely and had already provided essential advances in plant ion transport (e.g., MacRobbie and Dainty, 1958; Rains and Epstein, 1965; Epstein, 1966). The outward fluxes could be measured after loading plants with ^22^Na^+^ or ^42^K^+^/Rb^+^ and transferring then to different chemical solutions without the ions (e.g., Wang, 2006; Wang et al., 2006 for ^22^Na^+^ for roots of *Arabidopsis* and *Thellungiella*). Details and the methodical procedures are well described with different modifications to get more information about compartmentation of the absorbed ions and to exclude potential sources of errors (Cheeseman, 1986 for analysis of compartmentation based on eﬄux kinetics; Britto et al., 2006; Wang, 2006 for ^42^K^+^ in application for the tracer eﬄux by barley roots; Britto and Kronzucker, 2013 for comprehensive practical description of experimental procedures to measure potassium fluxes). The values of measured fluxes differ significantly, sometimes over 100 times depending on plant species, physiological conditions and ion concentrations (e.g., summarized for Na^+^ fluxes in: Kronzucker and Britto, 2011).

Analysis of unidirectional fluxes is often complemented by the other methods to obtain better understanding of the processes. An interesting comparison for influx of K^+^ (^86^Rb^+^) from 0.1 mM K_2_SO_4_ solution and net K^+^ influx measured by external K^+^ microelectrodes (prototype of MIFE) gave nearly the same values of fluxes: 2.6 μmole/(g FW^∗^h) and 2.5 μmole/(g FW^∗^h), correspondingly (Newman et al., 1987). Measurements of unidirectional ^24^Na^+^, ^42^K^+^ fluxes in barley together with membrane potential measurements and pharmacological profiling of the fluxes allowed to study high affinity transport of sodium from low μM – 50 mM solutions and provided predictions about possible mechanisms for the transport (Schulze et al., 2012).

Among methods to determine ion fluxes is the use of ion-selective fluorescent indicators for estimating cytoplasmic and vacuolar concentrations of Na^+^ and K^+^ and kinetics of their changes (e.g., mostly for protoplasts: Lindberg, 1995; Halperin and Lynch, 2003; D’Onofrio et al., 2005; Kader and Lindberg, 2005), measurements by intracellular ion-selective electrodes (e.g., Carden et al., 2003 and references there), ^23^Na-NMR spectroscopy (e.g., Bental et al., 1988) and several others; the methods are not discussed in detail here.

### Ion Concentrations in Cells

Ion concentrations are the reflection of net ion fluxes via plasma membrane. Certain range of ion concentrations, especially of K^+^, Na^+^, and Ca^2+^, is vital for cell physiology and function of proteins. Typical potassium concentrations in cytoplasm of plant cells were measured independently by several methods (including ion-selective electrodes, fluorescent dyes and X-ray microanalysis) and range around 60–140 mM (Pitman et al., 1981; Hajibagheri et al., 1988; Hajibagheri and Flowers, 1989; Walker et al., 1995, 1998; Korolev et al., 2000; Cuin et al., 2003; Halperin and Lynch, 2003; Shabala et al., 2006; Hammou et al., 2014), though concentrations above 200 mM were estimated by eﬄux analysis (reviewed in Britto and Kronzucker, 2008) and as low as 12 mM and even lower K^+^ concentrations were measured by ion-selective electrodes in root cells of potassium-deprived *Arabidopsis* plants (Armengaud et al., 2009). Higher potassium concentrations of 200–350 mM measured in cell sap by X-ray microanalysis or capillary electrophoresis are rather attributed to vacuolar compartment under sufficient potassium supply (Malone et al., 1991; Fricke et al., 1994; Bazzanella et al., 1998; Volkov et al., 2004). It is worth mentioning that in animal cells potassium seems to be among regulators of apoptotic enzymes activating them at K^+^ concentrations below 50 mM (Hughes and Cidlowski, 1999).

Sodium cytoplasmic concentrations of plant cells are usually low reaching about 20-50 mM after several days of NaCl treatment (Carden et al., 2001, 2003; Halperin and Lynch, 2003). Higher sodium concentrations had also been measured depending on duration of salt stress, external sodium, concentration of the other ions and on plant species: cytoplasmic sodium concentrations over 200 mM were reported in salt-tolerant halophytes (Hajibagheri and Flowers, 1989; Halperin and Lynch, 2003; Flowers and Colmer, 2008; Kronzucker and Britto, 2011).

An interesting example is halotolerant alga *Dunaliella salina*, which is a good unicellular eukaryotic model for studying salinity tolerance within the range of 0.05–5.5 M NaCl (e.g., Katz and Avron, 1985). Cytoplasmic sodium concentrations about 90 mM (88 ± 28 mM) were reported in the alga using ^23^Na-NMR spectroscopy (Bental et al., 1988). Na^+^ concentrations were nearly the same (within the error of a few measurements) in the algal cells adapted to a wide range of external Na^+^, from 0.1 to 4 M (Bental et al., 1988). Similar or even lower sodium concentrations below 100 mM were measured by the other methods for the alga under 0.5–4 M or 1–4 M sodium treatment (Katz and Avron, 1985; Pick et al., 1986). The small alga *D. salina* has length about 10–11 μm, width of 6 μm and volume around 200 fL (Masi and Melis, 1997) or even smaller dimensions with volume around 90–100 fL then (Katz and Avron, 1985). It probably possesses specific transport system to exclude Na^+^, similar to Na^+^-ATPase expected to function in the plasma membrane of the marine unicellular alga *Platymonas viridis* (Balnokin and Popova, 1994), alga *D. maritima* (Popova et al., 2005) and several other marine algae (reviewed in: Balnokin, 1993; Gimmler, 2000).

High sodium concentrations over 100 mM often have inhibiting effect on protein synthesis at least in salt-sensitive glycophytes (Hall and Flowers, 1973; Wyn Jones and Pollard, 1983; Flowers and Dalmond, 1992). Sodium is also (1) competing with potassium for allosteric sites of enzymes and (2) interacting with ion channels (for example, sodium ions change the gating of potassium outward rectifying currents in root protoplasts of halophyte plant *Thellungiella*, which are most likely carried by Shaker type potassium channels: Volkov and Amtmann, 2006). Moreover at the cellular level salt stress induces apoptosis (Katsuhara and Kawasaki, 1996; Huh et al., 2002; shortly reviewed in Shabala, 2009; Demidchik et al., 2010).

Much higher sodium concentrations could be tolerated in vacuoles, one of the functions of the organelle is to sequester and isolate sodium. Concentrations of sodium in vacuoles may exceed 0.5–1 M being up to ten times over the cytoplasmic sodium concentrations (eg Flowers et al., 1977; Zhao et al., 2005; Flowers and Colmer, 2008) at the expenses of activity of specific ion-transport systems (reviewed in: Martinoia et al., 2012). Under salt treatment of 2 M NaCl for 85 days shoot tissue concentrations of sodium in halophytes *Tecticornia* were about 2 M, so presumably vacuolar Na^+^ concentrations could be over 2 M in the halophytes (English and Colmer, 2013).

The reasons for sodium competing with potassium are that the ions have (1) the same electric charge, 1.6^∗^10^-19^ coulombs, (2) similar cation radii in non-hydrated, about 0.1 nm = 1.0 Å for sodium cation and 0.14 nm = 1.4 Å for potassium (diameter being nearly 2–3% of cell membrane thickness) and (3) hydrated forms, about 3.6 Å for sodium and 3.2 Å for potassium ions (Nightingale, 1959; Collins, 1997; Mähler and Persson, 2012) and, hence, (4) similar surface electric charge densities, which differ about two times (twice higher for non-hydrated sodium according to charge and diameter of the ion). Therefore interactions of Na^+^ and K^+^ with amino acids of protein surfaces, active centers of enzymes, pockets of allosteric regulation or binding of proteins, selectivity filters of ion channels are similar and often differ only several times in selectivity of the interactions. The selectivity depends on the nature and number of interacting amino acids and their spatial location. Molecular dynamics simulations together with conductivity measurements for several proteins and oligopeptides demonstrated up to five times higher affinity of sodium over potassium to the protein surfaces (especially with numerous carboxyl groups; Vrbka et al., 2006). The phenomenon could explain higher destabilizing effect of sodium over potassium on proteins (“salting them out”), which was initially discovered with white proteinaceous part of hen’s eggs by Hofmeister in 1888 (Hofmeister, 1888; Kunz et al., 2004). The effect could be the reason why potassium and not sodium is chosen and naturally selected for being the major intracellular monovalent cation, pumped into cells while pumping out sodium cations (Collins, 1997) though sea and ocean water contains more than 40 times higher concentration of sodium.

Under salt stress, for plants it is important to keep higher K^+^/Na^+^ ratio (Maathuis and Amtmann, 1999). It is essential, however, to mention that some proteins (due to specific amino acid composition or structural peculiarities) and processes from halophytes are able to withstand higher sodium concentrations without loss of activity (eg. Flowers and Dalmond, 1992; Premkumar et al., 2005); it seems to be the secondary evolutionary adaptation. It is also interesting that cell wall proteins of studied halophytes and also glycophytes did not change their activity within wide range of sodium concentrations, often from 0 to over 0.5–1 M (Thiyagarajah et al., 1996).

### Driving Forces and Pathways for Ion Transport to Cells

Transport of ions is driven by physico-chemical forces including differences of ion concentrations (to be more precise, activities of ions) and differences in electric potential at the sides of membranes.

**FIGURE 6:**
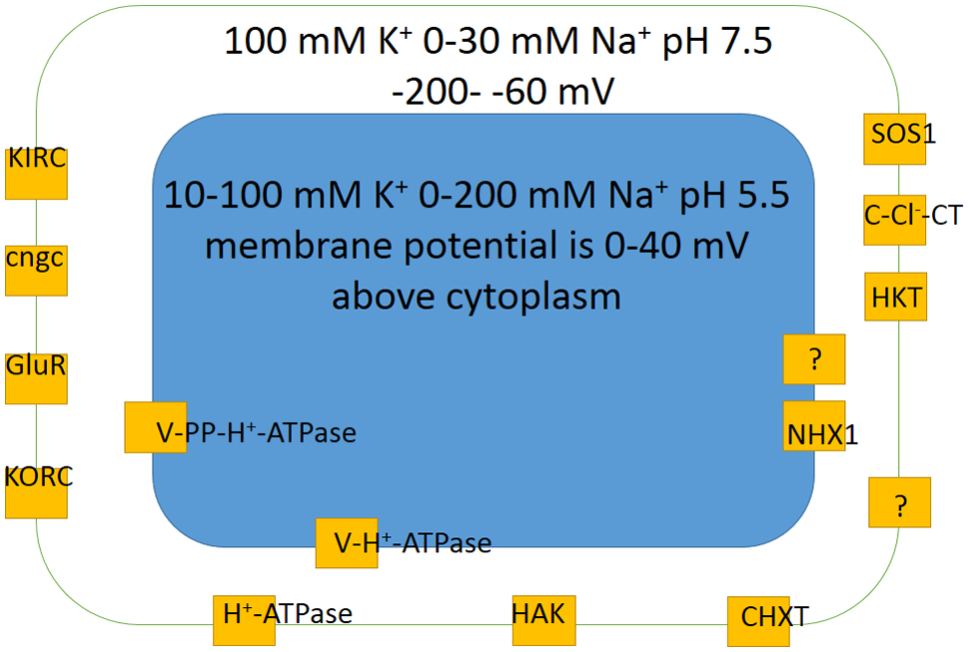
**Basic scheme of membrane potentials, potassium K^+^ and sodium Na^+^ concentrations and pH values in a generalized plant cell together with main ion transport systems ensuring potassium and sodium transport according to electrochemical forces.** Different cell types usually have less transporters, though specialized for more determined ion transport functions. The concentrations and membrane potentials are rather indicative and change depending on conditions of mineral nutrition and are not the same for different cell types (see text and references for more details). KIRC are inward rectifying potassium channels (e.g., Hirsch et al., 1998); KORC are outward rectifying potassium channels; GluR are glutamate receptors; cngc are cyclic nucleotide gated ion channels; HAK is high affinity potassium transporter; CHXT are cation H^+^ exchange transporters (e.g., Evans et al., 2012); HKT are high K^+^ affinity transporters; C-Cl^-^-CT are cation chloride contrasnporters; SOS1 is well studied sodium-proton antiporter; H^+^-ATPase is proton pump of plasma membrane; V-H^+^-ATPase is vacuolar proton pump; V-PP-H^+^-ATPase is vacuolar pyrophosphatase, another vacuolar proton pump; NHX1 is vacuolar sodium (cation)/proton antiporter. For more details and description see text.

Membrane potential of plant cells is routinely measured by microelectrodes with tiny sharp tips around 0.1 μm in diameter after impalement of a plant cell of interest (e.g., described in: Blatt, 1991). Recently developed voltage-sensitive fluorescent proteins and dyes (reviewed in: Mutoh et al., 2012) could also be used for at least indications of membrane potential in cell tissues and populations of cells (Matzke and Matzke, 2013). Membrane potentials below -70 mV and above -200 to -220 mV are recorded by microelectrodes though values around -300 mV were also reported; more negative values are often measured in root cells compared to leaf ones (apart from leaf guard cells) (Higinbotham, 1973; L’Roy and Hendrix, 1980; Blatt, 1987; Walker et al., 1995, 1998; Carden et al., 2001, 2003; Shabala and Lew, 2002; Fricke et al., 2006; Murthy and Tester, 2006; Shabala et al., 2006; Volkov and Amtmann, 2006; Armengaud et al., 2009; Hammou et al., 2014). Vacuolar membrane potential is the same or 10–40 mV above the values for cytoplasm with pH about 2 or over units lower, about 5.0–6.1 or less in vacuoles compared to pH = 7.0–7.7 in cytoplasm (e.g., Walker et al., 1995; Carden et al., 2003; Cuin et al., 2003; Martinoia et al., 2012) (**Figure 6**).

Thermodynamics of ion transport is described by several equations. Nernst equation applied to selectively permeable membrane links ion concentrations at the sides of membrane to the electric potential via the membrane under equilibrium conditions (when net flux of ions via the membrane is absent):

$$E_{S}=E_{1}{-E}_{2}=R*T/(Z_{S}*F)*ln([S_{2}]/[S_{1}])$$

(Hille, 2001). Here E is electric potential, R is universal gas constant equal to 8.31 J/(K^∗^mole), T is temperature in K, Z_S_ is the charge of ion S, F is Faraday constant equal to 96,500 s^∗^A/mole, [S_1_] and [S_2_] are concentrations of ion S at the sides of the membrane. Basically, the diffusion of ion S due to different concentrations is equilibrated by distinction in electric potential, which is about ± 60 mV (slightly depending on temperature) per 10-fold difference in concentrations with sign determined by the ion charge. For potassium with typical 100 mM in cytoplasm of epidermal root cell and low 100 μM in soil solution the membrane potential to ensure uptake of K^+^ should be below -180 mV to satisfy the electrochemically downhill transport of K^+^ ions. Lower concentrations of potassium outside the cells may require co-transport of K^+^ with the other ions (e.g., with H^+^).

For several ion species with specific permeabilities via the membrane a more complicated Goldman–Hodgkin–Katz voltage equation is applicable; it takes into account permeabilities of ions. For sodium, potassium and chloride (obviously more ions to be considered and more components should be added) the equation will be:

$$E_{\mathrm{membrane}}=R*T/F *ln((P_{\mathrm{Na}}{}_{+}{\left[{\mathrm{Na}}^{+}\right]}_{\mathrm{out}}+P_{K}{}_{+}{\left[K^{+}\right]}_{\mathrm{out}}{+P}_{Cl-}{\left[{\mathrm{Cl}}^{-}\right]}_{\mathrm{in}})/(P_{\mathrm{Na}}{}_{+}{\left[{\mathrm{Na}}^{+}\right]}_{\mathrm{in}}+P_{K}{}_{+}{\left[K^{+}\right]}_{\mathrm{in}}{+ P}_{\mathrm{Cl}}{}_{-}{\left[{\mathrm{Cl}}^{-}\right]}_{\mathrm{out}}))$$

where E_membrane_ is membrane potential via the membrane (or E_reversal_ with zero net ion current via the membrane), P are permeabilities of the corresponding ions and [] stands for concentrations of the ions (Hille, 2001). Usually potassium permeability is dominating and membrane potential is close to E_reversal_ of K^+^, though may change and depend on cell type. Active plasma membrane proton pump H^+^-ATPase (reviewed e.g., in: Palmgren, 2001) shifts membrane potential to more negative values compared to the calculated E_reversal_ for K^+^.

Transport of most ions including Na^+^ and K^+^ in plants occurs passively (following the electrochemical forces) via ion-selective proteinaceous pores of ion channels. Most ion channels can change their conformation from open to close states and vice versa (so called “gating”) under applied voltages or after binding ligands and regulators. Another pathway is via proteinaceous transporters with slower transport rates. Ion transport via ion channels is electrogenic since ions carry electric charge, while transporters realize electrogenic or non-electrogenic transport, transporting one ion or co-transporting/antiporting several charged ions in one or opposite directions, correspondingly.

**FIGURE 7:**
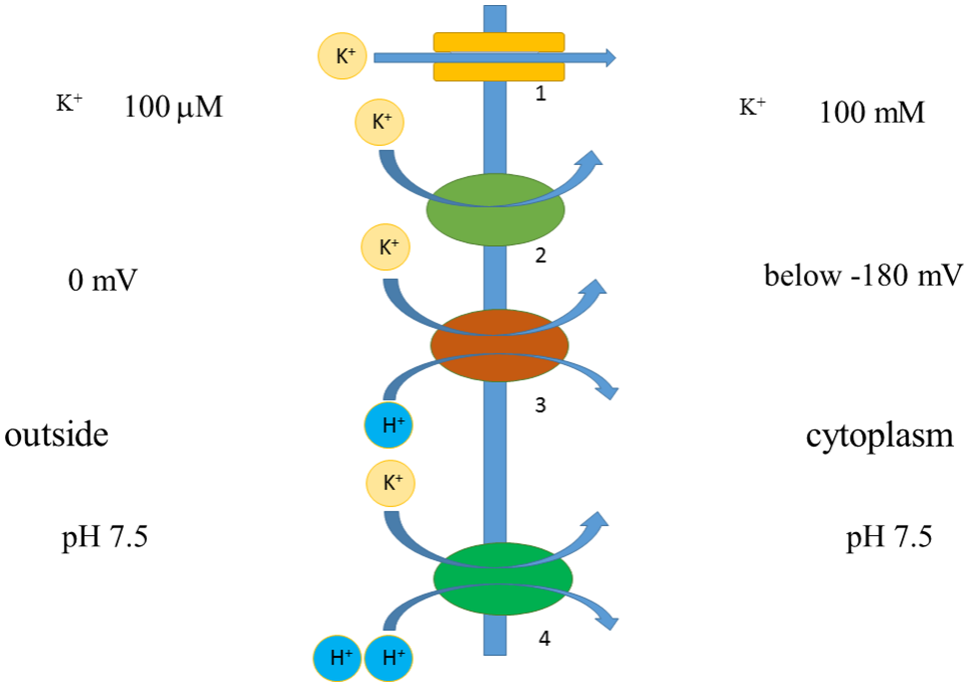
**Simplified scheme demonstrating principles of ion transport via membrane.** Voltage difference below -180 mV allows potassium transport against 1000-fold concentration difference via potassium-selective pore of ion channel 1. Similar thermodynamically favorable potassium transport with lower rates and specific mechanism is facilitated by potassium transporter 2. Transporters 2 and 3 are H^+^/K^+^-cotransporters, they co-transport one or two H^+^ per K^+^; H^+^ is transported according to voltage difference, hence adding energy for K^+^ transport. Transporters 2 and 3 can potentially lead to inward transport of K^+^ against over 1,000,000 concentration difference; their functioning depends also on pH difference across membrane. More details are in the text.

Co-transport of several ions or even small molecules may add extra energy for transport (**Figure 7**). For example, HAK transporters presumably co-transport K^+^ together with H^+^ (Banuelos et al., 1995; Rodriguez-Navarro, 2000; Grabov, 2007), which gives several orders of concentration differences extra due to transport of H^+^ according to electric charge. Membrane potential of -180 mV potentially allows potassium uptake, when co-transported with H^+^ with, e.g., stoichiometry 1:1 under similar external pH to pH of cytoplasm, against 10^6^ differences in K^+^ concentrations (e.g., Rodriguez-Navarro, 2000). Higher concentrative capacity could be achieved using also pH differences or higher number of protons per K^+^; cytoplasmic pH is about 7.5 and external low pH of 4 will add over three orders of concentration more. The surprising example is described for yeast *Schwanniomyces occidentalis*, which was reported to deplete external potassium to 0.03 μM, presumably taking up K^+^ against 3,000,000 differences in concentrations (assuming over 100 mM of cytoplasmic K^+^) due to HAK transporters (Banuelos et al., 1995). Much higher concentrations, around 80 μM, arising from K^+^ contamination from agar and the other chemicals (Armengaud et al., 2009; Kellermeier et al., 2014) (though contamination from agar was estimated at 1–3 μM: Kellermeier et al., 2014) resulted in symptoms of severe potassium deficiency in *Arabidopsis* and essentially changed transcription profile in roots and shoots of the plants (Armengaud et al., 2004), so more detailed examination with special attention to transport systems of different species (e.g., Coskun and Kronzucker, 2013) is required. Potentially new transporters and ion channels from different organisms could be a source of diversity and comparison for the existing pool of membrane transport proteins and for creating novel artificial transporters.

A set of transporters and ion channels is specific for cell types and organisms, includes tens and more distinct characterized so far proteins, which often form heteromers with variable properties and regulation (**Figure 6**; e.g., reviewed in: Isayenkov, 2012). Detailed analysis of genome sequences of salt-sensitive model plant *Arabidopsis* revealed that about 5% of about 25,000 genes of the plant potentially encode membrane transport proteins; the genes of about 880 proteins are classified in 46 unique groups, while genes of cation channels/transporters predict for coding over 150 proteins (Mäser et al., 2001). Special databases include information about transport proteins, e.g., plant membrane transport database http://aramemnon.uni-koeln.de/; http://www.yeastgenome.org/ is a useful source of information for yeast proteins including yeast membrane transport proteins.

### Cell Size-Volume-Surface/Volume Ratio and Effects of Cell Wall

**FIGURE 8:**
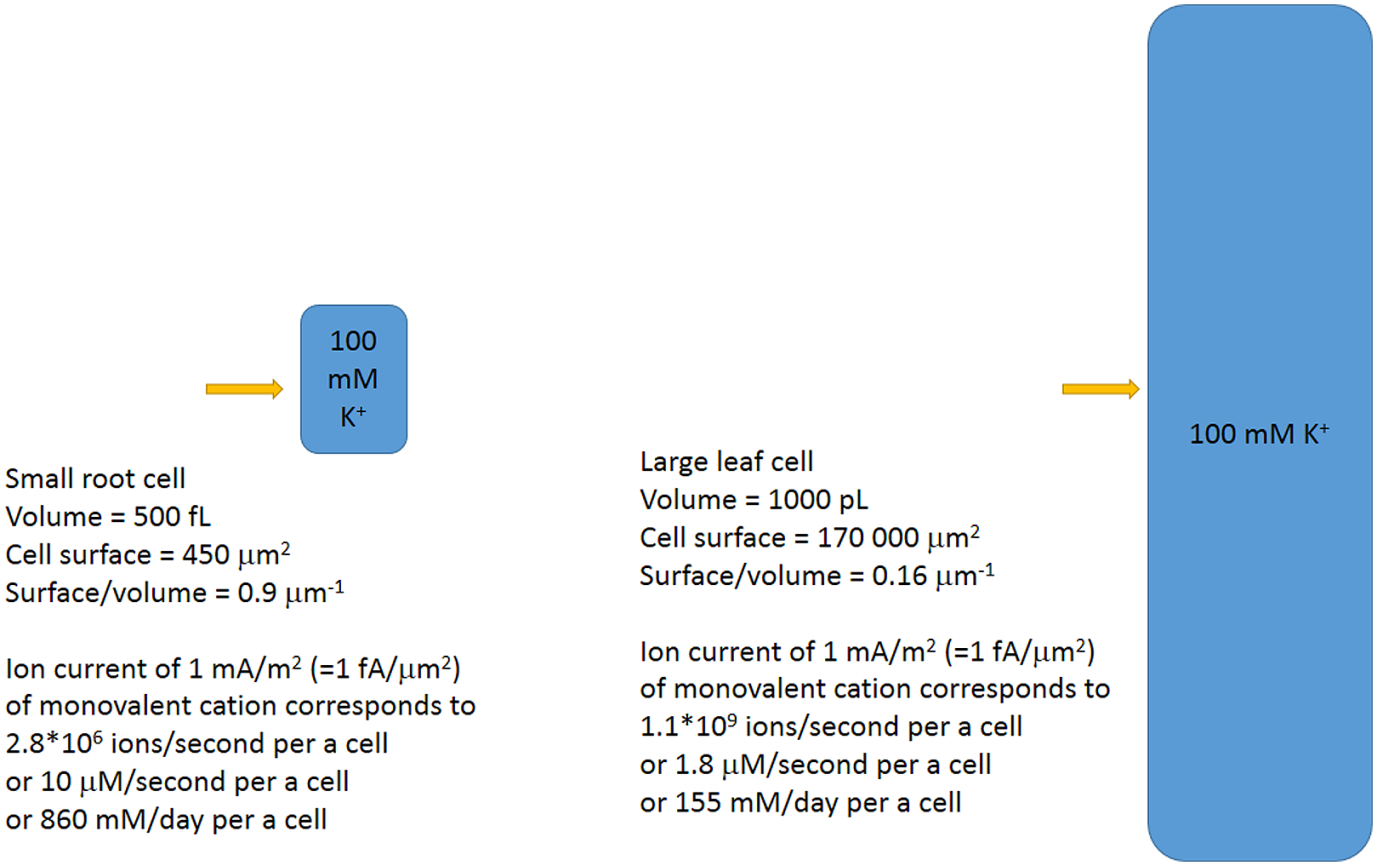
**Simplified scheme of different plant cells and the ion fluxes for the cells.** The same ion fluxes result in different changes of ion concentrations for cells of different sizes and surface/volume ratios. Ion fluxes are directly recalculated from number of ions/second to concentrations.

Interestingly, cell surface/volume ratio has an effect on ion concentration under the same ion fluxes (**Figure 8**). The sizes of cells within a plant differ orders of magnitude. Cells of xylem parenchyma in roots of dicot *Arabidopsis* are less than 5 μm in diameter with length often below 20–30 μm (eg: Dolan et al., 1993; Ivanov, 1997; Kurup et al., 2005; Verbelen et al., 2006; Ivanov and Dubrovsky, 2013); the cells could be isolated and result in protoplasts of about 10 μm in diameter compared to larger 20 μm epidermal protoplasts from root elongation zone or 15–25 μm protoplasts from root cortex parenchyma cells and the other root tissues (Demidchik and Tester, 2002; Demidchik et al., 2002a; Volkov and Amtmann, 2006). The volumes would correspond to 4/3^∗^π^∗^R^3^ that is about 500 fL for 10 μm protoplasts and about 8 pL for 25 μm protoplasts.

Cells in leaf epidermis of several monocots are quite large. Barley epidermal leaf cells could be up to 2 mm long and about 25–30 μm wide with nearly square cross-section, thus reaching volume over 1000 pL (Volkov et al., 2007). Several studies involved isolated protoplasts from barley leaf epidermal cells and reported 60 μm (100 pL) protoplasts (Dietz et al., 1992), 40 μm (from 20 to 80 μm) (30 pL, from to 4 to 250 pL) protoplasts (Karley et al., 2000), 25 pL protoplasts with large variations (over 10 times) in volume (Volkov et al., 2007). It is worth mentioning that about 99% of large epidermal leaf protoplasts could be occupied by vacuole (Winter et al., 1994).

Assuming volumes of usual plant cells within 500 fL to 1000 pL, the calculated surface to volume ratios will differ about 10 times for the cells: from about 0.9 μm^-1^ to 0.16 μm^-1^ for oblong cells within plant tissues. The larger cells need higher ion fluxes or larger time for the same concentration changes.

**FIGURE 9:**
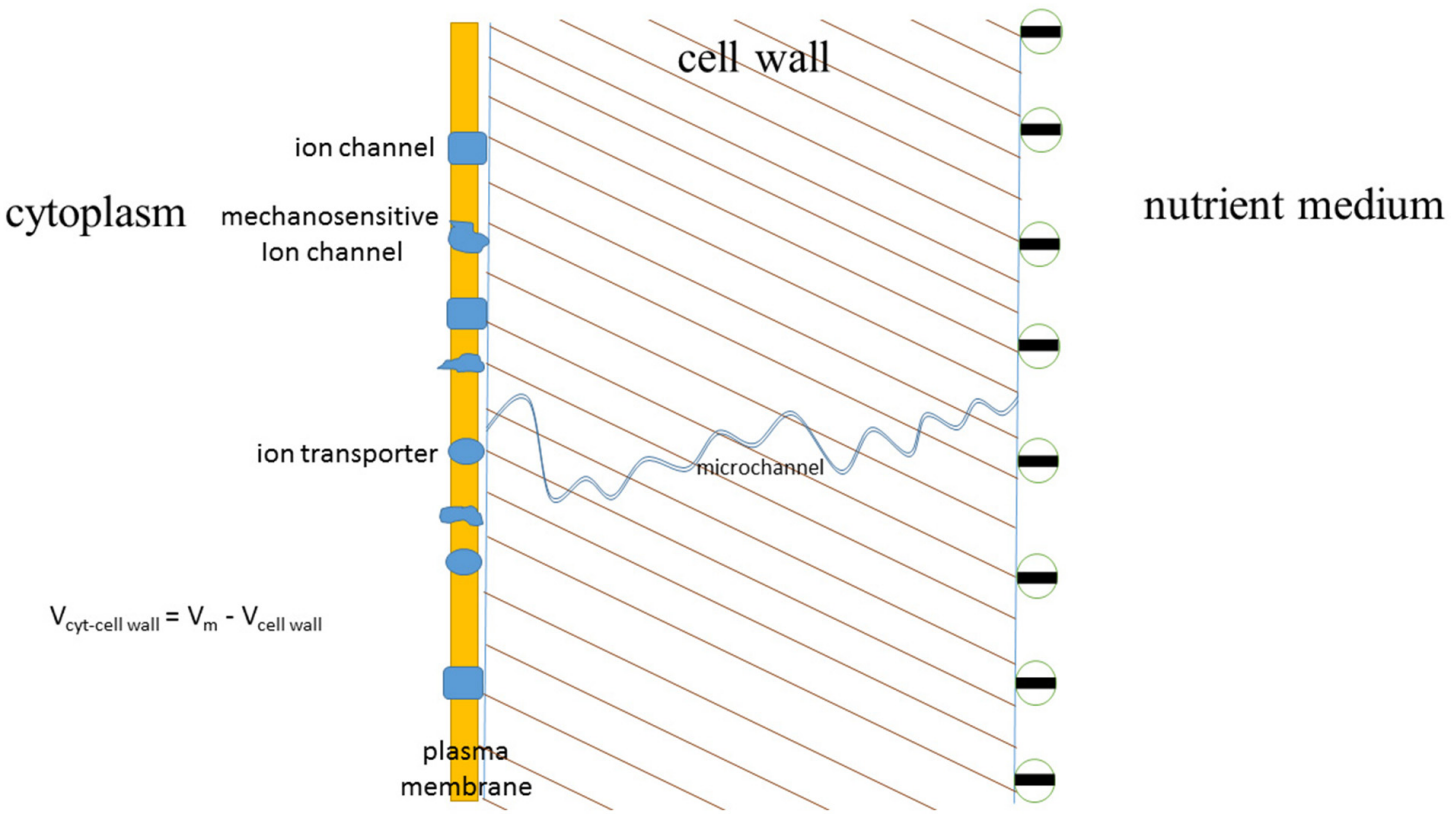
**The scheme indicating expected and studied effects of cell walls on ion fluxes and cell plasma membrane potential.** Plasma membrane of approximately 10 nm thickness is surrounded by the cell wall of about 250 nm thickness, the interior of the cell is under hydrostatic turgor pressure of several bars (1 bar = 0.1 MPa) due to concentration differences and, hence, differences in osmotic pressures inside the cell and outside of it. Cell wall has negative surface charge at pH around 6–7, hence influencing the voltage difference between the sides of plasma membrane. One of micro- or nanochannels in the porous cell wall is depicted with charged inner walls and potential ion-rectifying properties. More details are given in the text.

Effects of cell walls on ion transport are well known though not often remembered and studied in detail (**Figure 9**). They include changes of cell membrane potential depending on ion composition of ambient medium due to fixed electric charges in cell walls, assumed ion-rectifying properties of cell walls and expected effect of shrinking and swelling of cell walls on mechanosensitive ion channels. Effects of cell walls on ion buffering and ion concentrations have more experimental evidence. For example, protoplasts isolated from bean leaf mesophyll did not show NaCl-induced calcium eﬄux compared to mesophyll tissue (Shabala and Newman, 2000). Salt-induced H^+^ eﬄux also differed between protoplasts compared to tissue, so, presumably, all the Ca^2+^ eﬄux over an hour of measurement was from calcium ion exchange stores of cell walls (Shabala and Newman, 2000).

### Ion Channels vs. Ion Transporters: More about Pathways of Ion Transport to Cells

One of disputable questions of ion transport is about the relative role of ion channels and transporters in transport. It is commonly accepted that ion channels in an open state/conformation allow passage of over 10^6^–10^8^ ions per second via a selective pore formed within a protein molecule. The diameter of the pore is determined by the molecular structure of ion channel, from 12 Å for potassium channel KcsA with narrow part of 4 Å in diameter (e.g., Doyle et al., 1998; Jiang et al., 2002; MacKinnon, 2004) to 15 Å and even 28 Å diameters of pores for the general bacterial porins with low selectivity and permitted passage for small hydrophilic molecules (about 6 Å pores for the highly selective porins) (e.g., Galdiero et al., 2012). The diameter of the pore and amino acids lining it essentially determine the ion selectivity of ion channel and potential number of passing ions per unit of time. The selectivity could be, for example, over 1,000 for K^+^ over Na^+^ in potassium selective ion channels or over 10 for Na^+^ over K^+^ in sodium selective channels due to special selectivity filters with conserved amino acids for specific channel types. Often amino acid sequence glycine-tyrosine-glycine (GYG) indicates selectivity for K^+^, introducing mutations into the pore to change the amino acids converted potassium selective ion channels to non-selective ones (Heginbotham et al., 1992). The interactions of ions with the protein molecule of ion channel are not well understood yet and probably involve non-electrostatic ion–ion interactions, van der Waals forces, interaction with water molecules and numerous other interactions. Several methods of modeling and simulations of molecular dynamics are applied within at least the last 30 years; sharp increase in computing power allowed to include the lipid environment of membranes, pH and the known biochemical factors and regulators to the models (e.g., reviewed in: Maffeo et al., 2012).

**FIGURE 10:**
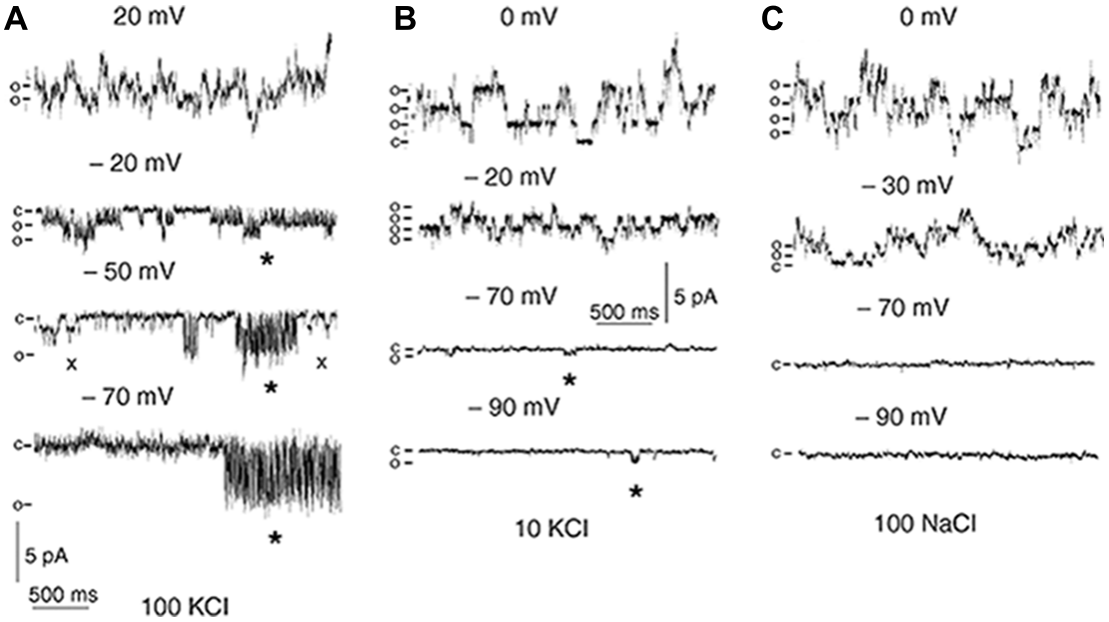
**Single-channel recordings in outside-out patches of *T. halophila* root protoplasts.** The pipette solution was 100 mM KCl. The bath solution contained 100 mM KCl **(A)**, 10 mM KCl **(B)** or 100 mM NaCl **(C)**. c, current level with no open channels; o, current levels of single-channel openings. Spiky openings of outward-rectifying channels allowing inward current are indicated with asterisks. Openings of a second type of channel are indicated with crosses. Reproduced from Volkov and Amtmann (2006) with the permission from the publisher John Wiley and Sons.

Direct measurements are the basement for investigating ion fluxes via ion channels; they provide information about permeating ions, number of ions per second, selectivity and complex transitions of protein molecules of ion channels (gating) during the transport processes. Indeed, a small current of 1 pA corresponds to 10^-12^ A/(1.6^∗^10^-19^ coulombs) ≈ 6^∗^10^6^ ions/second (1.6^∗^10^-19^ coulombs is elementary charge, a charge of monovalent cation), while most ion channels demonstrate much larger electric currents with complex voltage-dependent patterns of open–closed states (**Figure 10**).

Transporters could be considered as enzymes where conformational changes of a protein molecule are required for a complete transport cycle of ions (e.g., Gadsby, 2009). Turnover rate of the transporter is the number of complete transport cycles performed per second (e.g., Longpré and Lapointe, 2011). The lower estimate for turnover rate of transporters is the activity of ion pumps. Plant plasma membrane H^+^-ATPase pumps about 100 ions per a second (Sze et al., 1999). The value is comparable to 20–100 H^+^ per second by yeast plasma membrane ATPase Pma1 (Serrano, 1988) and turnover rate of 160 s^-1^ of animal Na^+^/K^+^-ATPase (Skou, 1998). Similar or even lower turnover rates, from 3 to 60 s^-1^, were shown for sodium/glucose cotransporter (Longpré and Lapointe, 2011 and references therein), while turnover rate about 500 s^-1^ was estimated for sucrose/H^+^ co-transporter from maize ZmSUT1 (Carpaneto et al., 2010). The highest possible turnover rate for activity of ion transporters could be assessed from protein structure studies and frequency of conformational changes with estimated upper limit of 10^6^ s^-1^ (Chakrapani and Auerbach, 2005), which seems to be an overestimated value. A more realistic value for the fast transporters is around 10,000 ions/second, when they are mutated and have accelerated turnover rate (Gadsby, 2009). A higher value of 100,000 ions per second was reported for Cl^-^/H^+^ antiporter ClC-5, which is rather a unique type of transporter similar to ClC channels (Zdebik et al., 2008).

Mechanisms of ion transport by transporters are less understood than transport via pores of ion channels, though the genes of transporters are sequenced and well-studied. Briefly, several mechanisms are expected for different transporters and described below.

HKT transporters with at least eight transmembrane domains could be similar to ion channels, they form a specific ion-selective pore with properties distinct from the pore of ion channels according to basic crystal structure analysis (Cao et al., 2011; reviewed in: Yamaguchi et al., 2013). HKT transporters can electrically resemble ion channels with similar IV curve, reversal potential of ion current mediated by HKT can shift following ion concentrations inside and outside the cell. The phenomenon is observed in *Xenopus* oocytes heterologously expressing different HKT transporters (Jabnoune et al., 2009; Almeida et al., 2014; de Almeida, 2014), where rectification and Nernstian shift (according to Nernst equation for ion concentrations and voltages) in reversal potential were measured. Pore of HKT transporters has selectivity filter in the first transmembrane domain with conserved glycine for K^+^-selective and serine for Na^+^-selective HKT transporters (reviewed in Yamaguchi et al., 2013; de Almeida, 2014). An extra amino acid constriction with arginine residue in the last transmembrane domain makes an additional energy barrier for ion transport (Kato et al., 2007; Cao et al., 2011; reviewed in: Yamaguchi et al., 2013; Benito et al., 2014; de Almeida, 2014). Selectivity of HKT transporters for Na^+^ or K^+^ could be altered by amino acid substitutions, while K^+^/Na^+^ symport or Na^+^ uniport are exhibited by different HKT transporters and even varied from K^+^/Na^+^ symport to Na^+^ uniport depending on the cation concentrations (Mian et al., 2011; Almeida et al., 2014; reviewed in Almeida et al., 2013; Waters et al., 2013; de Almeida, 2014). Some HKT transporters symport K^+^ with Na^+^ at low K^+^ concentrations (reviewed in Waters et al., 2013); presumably Na^+^ ion adds energy for co-transport “pushing” K^+^ via the amino acid constrictions (Benito et al., 2014), though the exact mechanisms of transport are still to be elucidated.

HAK transporters are not found in animals and Protista (Grabov, 2007). Presumably they co-transport K^+^ together with H^+^ (Banuelos et al., 1995; Rodriguez-Navarro, 2000; Grabov, 2007), but had not been crystallized so far while attempts to express them in Xenopus oocytes failed; therefore mechanisms of ion transport by HAK transporters are not well studied yet. Gene sequences and comparison of HAK transporters predict for 10–14 transmembrane domains (Greiner et al., 2011). Amino acid substitutions within the region between the second and third putative transmembrane domains of *Arabidopsis* HAK5 transporter essentially changed ion transport selectivity indicating for selectivity filter within the region (Alemán et al., 2014). Lack of putative specific pore similar to HKT transporters suggests that HAK transporters realize specific mechanism for K^+^/H^+^ symport (Alemán et al., 2014).

Nhx1 and SOS1 are cation/H^+^ antiporters. SOS1 has 12 predicted transmembrane domains at the N-terminal part and long C-terminal tail composed of 700 amino acids (Shi et al., 2000), the protein forms homodimers (Núñez-Ramírez et al., 2012). Molecular mechanisms of Na^+^/H^+^ antiport by SOS1 are under investigation, though the known crystal structure of bacterial Na^+^/H^+^ antiporter NhaA (Hunte et al., 2005) could be a potential basement for understanding transport by both Nhx1 and SOS1. NhaA has 12 transmembrane domains, exists as a dimer, amino acid helices of the protein form negatively charged funnel-like structure, which leads to cytoplasm from the center of membrane and selectively attracts cations. Cation-bound Nha1 follows transformation and releases cation to the outer side being protonated at the same time at aspartate moieties. Deprotonation, release of H^+^ to cytoplasmic side and return to the initial conformation completes the transport cycle (Hunte et al., 2005). The mechanism with conformation changes limited within a part of the protein makes NhaA one of the fastest transporters; NhaA has activity of catalytic center (turnover rate) about 89,000 s^-1^ (reviewed in Hunte et al., 2005).

Tonoplast Nhx1 could share the same transport mechanism. Discovery of Nhx1 initially provided a molecular basis for Na^+^/H^+^ antiport activity of vacuolar membrane. Na^+^/H^+^ antiport was measured in tonoplast of several plants: it included amiloride-sensitive transport in vesicles from *Beta vulgaris* (Blumwald and Poole, 1985) and from mesophyll cells of halophytic plant *Mesembryanthemum crystallinum* (Barkla et al., 1995), vesicles from roots of NaCl-grown salt-tolerant *P. maritima*, but not salt-sensitive *P. media* (Staal et al., 1991), in preparations from salt-grown barley roots (Garbarino and DuPont, 1988). Gene *AtNHX1* of the transporter was cloned from *Arabidopsis* and rescued some of the salt-sensitive yeast phenotypes under heterologous expression (Gaxiola et al., 1999). Moreover, overexpression of *AtNHX1* conferred salinity tolerance to *Arabidopsis* and significantly increased Na^+^/H^+^ antiport activity of vacuolar membrane (Apse et al., 1999). Expression of *AtNHX1* in yeast resulted in increased amiloride-sensitive electroneutral Na^+^/H^+^ exchange in yeast vacuolar vesicles (Darley et al., 2000). However, AtNHX1 with 9–11 putative transmembrane domains (reviewed in Rodríguez-Rosales et al., 2009) demonstrated also high K^+^/H^+^ exchange capacity depending on regulation by luminal C-terminal domain; the ratio of maximal rates of K^+^ to Na^+^ transport rose upon binding calmodulin in calcium and pH-dependent manner (Yamaguchi et al., 2003, 2005). Further evidence for role of *AtNHX1* in K^+^ transport came from transgenic plants. Overexpression of *AtNHX1* in tomato plants conferred higher vacuolar K^+^ under different growth conditions and increased salinity tolerance via retaining intracellular K^+^ without influencing vacuolar Na^+^ accumulation (Leidi et al., 2010). The simple model of AtNHX1 transporting and localizing excess Na^+^ in vacuole was modified to more complex schemes. It was suggested that AtNHX1 is more important for K^+^ transport to vacuole thus stimulating K^+^ uptake by roots, then K^+^ ions recycle between cytoplasm and vacuole, while Na^+^ is transported to vacuole and “locked” there (Jiang et al., 2010). Different effects of overexpressed *NHX* genes on vacuolar K^+^ and Na^+^ concentrations under salt stress and increase in salt tolerance led to conclusions that NHX plays role in both Na^+^ and K^+^ vacuolar transport and K^+^ homeostasis (reviewed in: Rodríguez-Rosales et al., 2009; Bassil et al., 2012; Yamaguchi et al., 2013; Bassil and Blumwald, 2014).

Unfortunately, ion fluxes via a single transporter (order of several fA or much lower) are below the resolution for electrophysiological measurements. However, potentially the ion currents via at least hundreds and rather thousands or millions of electrogenic ion transporters could be measured under specific conditions. A report about unitary conductance of Cl^-^/H^+^ antiporter ClC-5 is an exception, the conductance of 0.45 pS (ion current about 63 fA at 140 mV) for the transporter was determined from noise analysis of recordings with hundreds/thousands of transporters (Zdebik et al., 2008).

To study their properties, transporters are routinely heterologously expressed in large *Xenopus* oocytes. Detectable ion currents or fluxes of radioactive tracers are reported following the expression at the background of usually small endogenous electric currents of the oocytes. The successful expression in *Xenopus* oocytes was reported for several HKT transporters and cation-chloride cotransporters, attempts to record activity of HAK, SOS1, or Nhx transporters were less fruitful so far (Rodriguez-Navarro, 2000; Liu et al., 2001; Colmenero-Flores et al., 2007; Jabnoune et al., 2009; Rodríguez-Rosales et al., 2009). Mature *Xenopus* oocytes used for heterologous expression are quite even and have diameter of 1 mm, so measurements could be easily normalized per surface area. A typical surface area of *Xenopus* oocyte is 4^∗^π^∗^500^∗^500 μm^2^ ≈ 4^∗^3.14^∗^250000 ≈ 3,100,000 μm^2^. Assuming that the very high recorded values of about -10 μA at -150 mV for heterologous expression of rice transporter HKT in *Xenopus* oocytes (Jabnoune et al., 2009) are reasonable and not due to incorrect folding/partial proteolysis/interaction with endogenous transport systems of *Xenopus* oocytes, we can recalculate the current per a unit of oocyte surface and compare with recordings from plant protoplasts: -10 μA/3,100,000 μm^2^ ≈ 3 pA/μm^2^ = 3,000 mA/m^2^ or about 1 nA per a small root protoplast with diameter around 10 μm. The values are very high and comparable or even much higher (see below) than the ones recorded from activity of ion channels using patch clamp.

Another theoretical estimate is useful for assessing activity of ion transporters expressed in *Xenopus* oocytes. Assuming the high turnover rate for the transporter around 10,000 and the very high expression of about 10,000 transporters/μm^2^ (means a transporter per 10^∗^10 nm^2^, nearly maximal density due to the physical and steric limitations) we get the possible presumed current per oocyte of *Xenopus*: 10,000 ions per transporter per second ^∗^ 3,100,000 μm^2^^∗^ 10,000 transporters/μm^2^ = 3,1^∗^10^14^ ions/second, then taking elementary charge of monovalent ion being 1.6^∗^10^-19^ coulombs, the estimate gives 1.6^∗^10^-19^ coulombs/ion ^∗^ 3,1^∗^10^14^ ions/second ≈ 50 μA per an oocyte. An excessive order of magnitude could be reasonably explained by a lower level of expression and lower transport rate of a transporter, so gives a reasonable agreement with experimental data and leads to several conclusions.

(1) Transporters can provide sufficient ion currents for registered ion transport under conditions of salinity.

(2) Ion channels are not the only essential pathway for ion transport under salinity.

(3) Balance between relative share of ion transport by ion channels or by ion transporters depends on abundances of the corresponding proteins (ion channels or transporters), their regulation and the other factors (composition of ion solutions, membrane potential etc.). Transporters can provide coupled transport of several ions and potentially may ensure fine-tuning of ion transport, while ion channels provide large ion fluxes when required.

The estimates indicate that total ion current via thousands/millions of electrogenic transporters could be measured and characterized in plant protoplasts using patch clamp in whole-cell configuration (recording sum of all ion currents via the whole membrane). Well-studied non-selective cation channels with low conductance carry small instantaneous currents; potential total current via numerous transporters could be of the same range. Ion current via ion-selective ion channels is described by Goldmann–Hodgkin–Katz equations based on assumption of independent passage of ions via channel pore (or constant electric field along the diffusion zone) (Hille, 2001), additional charges of lipid bilayer and the surface of ion channel can modify the ideal curves (Alcaraz et al., 2004). However, similar to Goldmann–Hodgkin–Katz curves were recorded for HKT transporters expressed in *Xenopus* oocytes (Jabnoune et al., 2009; de Almeida, 2014).

Recently sodium currents via AtHKT1;1 transporters were presumably measured in *Arabidopsis* root stelar protoplasts overexpressing AtHKT1;1 (Møller et al., 2009; Xue et al., 2011). Patch clamp experiments recorded about 30 mA/m^2^ lower (more negative) currents in 10 mM external Na^+^ and about 50 mA/m^2^ lower currents in 25 mM external Na^+^ at -120 mV, when compared to control protoplasts. The currents in AtHKT1;1 overexpressing protoplasts shifted the reversal potential according to external Na^+^ concentrations, so confirmed Na^+^ selectivity. Further study (Xue et al., 2011) compared Na^+^ and K^+^ currents in root stelar cell protoplasts from wild type (control) and *athtk1;1–4* mutant plants lacking AtHKT1;1. Potassium currents were similar (about -50 pA at -120 mV in 5 mM internal/50 mM external K^+^), while sodium currents were about -50 pA at -120 mV in control protoplasts at 50 mM internal/50 mM external Na^+^ compared to about -10 pA in *athtk1;1–4* mutant ones and demonstrated Nernstian shift according to reversal potential of sodium (Xue et al., 2011). The results pose questions for future study. Earlier it had been demonstrated that non-selective cation currents in similar root protoplasts of *Arabidopsis* (e.g., Demidchik and Tester, 2002 and see below) are slightly (1.5 times) more selective for potassium over sodium, so predictably *athtk1;1–4* mutant protoplasts should have 2–4 times higher sodium currents than measured in (Xue et al., 2011) due to expected non-selective cation currents.

The non-selective cation currents are studied well for root protoplasts, especially in *Arabidopsis* and carried by cyclic nucleotide gated channels (about 20 genes for *Arabidopsis*) and glutamate receptors (about 20 genes for *Arabidopsis*) (see below). One of the possible explanations for the paradox is to assume that nonselective cation currents in root stelar protoplasts of *Arabidopsis* are highly selective for potassium over sodium; the selectivity was shown for root protoplasts of *Thellungiella* (Volkov and Amtmann, 2006), salt-tolerant relative of *Arabidopsis* (Bressan et al., 2001; Inan et al., 2004; Amtmann, 2009). It is important to understand specific tissue and cell type expression of genes and proteins for non-selective cation channels and HKT transporters for characterizing their role in total ion currents. So far, more electrophysiological studies were performed with non-selective cation currents, while HKT transporters were mostly studied using molecular biology with the known genes in heterologous expression systems.

Special modeling for different pores of ion channels will help to understand better the peculiarities of ion currents via ion channels and HKT-like transporters. Pharmacological analysis and profiling of ion currents is also essential together with the further use of mutants (knock out or overexpression) and heterologous expression of the genes of interest in cell culture or in *Xenopus* oocytes. Non-selective ion currents are well characterized electrophysiologically and pharmacologically, especially for root protoplasts (White, 1993; White and Lemtiri-Chlieh, 1995; Roberts and Tester, 1997; Tyerman et al., 1997; Tyerman and Skerrett, 1998; Demidchik and Tester, 2002; reviewed in: Demidchik et al., 2002b; Volkov and Amtmann, 2006; reviewed in: Demidchik and Maathuis, 2007), less is known for transporters. Recently quinine (500 μM) was shown to have slight inhibiting effect on ion currents induced by HKT1;4 transporters from durum wheat after heterologous expression in *Xenopus* oocytes; Zn^2+^, La^3+^, Gd^3+^, or amiloride had no effect (Ben Amar et al., 2014). For cation-chloride co-transporter from *A. thalina*, which was expressed in *Xenopus* oocytes and presumably transported Na^+^:K^+^:2Cl^-^ 100 μM bumetanide had an inhibiting effect on uptake of radioactive ions similar to analogous animal co-transporters (Colmenero-Flores et al., 2007). Amiloride is shown to inhibit Nhx1 vacuolar Na^+^(cation)/H^+^ antiporter (Barkla et al., 1990; Darley et al., 2000). Experiments with heterologous expression of rice HKT transporter OsHKT2;4 in *Xenopus* oocytes demonstrated channel-like behavior with single channel traces and inhibition by Ba^2+^, La^3+^, and Gd^3+^ (Lan et al., 2010), however, the properties were not typical for a transporter and further on the results were not confirmed and attributed to endogenous currents of the expression system (Sassi et al., 2012).

A further complication for understanding the pathways of membrane ion transport comes from electroporation experiments (e.g., Pakhomov et al., 2009; Wegner et al., 2011, 2013; Wegner, 2013, 2014). Nanopores with diameter about 1.0 -1.8 nm are formed in lipid membrane bilayer of several animal cell lines and plant protoplasts after (1) voltage pulses of 1 V and lower to -350 to +250 mV and (2) also during patch clamp experiments after applied holding voltages above +200–250 mV or below -300 to -250 mV (Pakhomov et al., 2009; Wegner et al., 2011, 2013; Wegner, 2013). The voltage-induced nanopores existed for minutes and demonstrated selectivity for cations (including even TEA^+^) over anions (Cl^-^) and slight selectivity for different cations (Ca^2+^ and Li^+^ were the most permeable); certain similarity to behavior of non-selective cation channels was found (Wegner et al., 2011, 2013; Wegner, 2013). It is a question whether the nanopores appear under physiological conditions in plants (voltages below -300 mV were recorded for plant cells). Heterologous expression of specific ion channels together with use of mutants, elucidating detailed properties, pharmacological profiles and peculiarities of ion fluxes are required to exclude misinterpretation.

## Genetic Engineering with Non-Specific or Tissue-Specific Overexpression/Knockout/Disruption of Specific Transporters Modifies Salinity Tolerance

Several obvious ways to achieve salinity tolerance include: (1) decreasing sodium conductance and increasing potassium/sodium selectivity of plasma membrane of root epidermal cells; (2) increasing sodium eﬄux by root epidermal cells; (3) increasing sodium accumulation in vacuoles; (4) altering sodium and potassium loading and unloading to xylem and phloem depending on plant strategy to cope with salinity. The strategies had been realized by salt-tolerant plants or revealed in plants overexpressing genes of specific transporters.

Modification of gene activity started after the essential rise and success of molecular methods together with the identification and characterisation of individual ion channels and transporters. Lower sodium conductance and higher K^+^/Na^+^ selectivity of root epidermis discovered in halophytes (see above) could be potentially reached in agriculturally important plants by RNA silencing of non-selective cation channels or modifying their expression pattern and regulation. However, still not much is known about the exact genes for the ion channels and importance of the individual genes in sodium uptake. Successful attempts to overexpress or knockout genes of vacuolar proton pump H^+^-PPase, NHX, HKT, or SOS1-like transporters and to modulate the salinity tolerance of plants had already been reported.

Overexpression of the vacuolar H^+^-pump would enhance the proton pumping activity at vacuolar membrane and thus permit to accumulate more Na^+^ in vacuoles due to activity of Na^+^(cation)/H^+^ antiporters NHX. The choice of H^+^-pyrophosphatase is explained by a single gene required for the protein, while the other vacuolar H^+^-ATPase is composed of several subunits and needs correct overexpression of several genes (reviewed in e.g., Silva and Gerós, 2009). Overexpression of vacuolar H^+^-PPase under control of strong non-specific viral *35S* promoter sharply increased salinity tolerance in *Arabidopsis*, to 250 mM of NaCl (Gaxiola et al., 2001). Overexpressing plants accumulated more sodium and potassium in their leaves and also demonstrated higher drought resistance. Further attempts to overexpress vacuolar H^+^-PPases from different microbial (D’yakova et al., 2006) and plant species increased salinity tolerance in tobacco (D’yakova et al., 2006; Gao et al., 2006; Li et al., 2014), transgenic rice overexpressing also vacuolar transporter NHX1 (Zhao et al., 2006), in alfalfa (Bao et al., 2009), cotton (Pasapula et al., 2011), tomato (Bhaskaran and Savithramma, 2011), and sugarcane (Kumar et al., 2014). The experiments used *35S* promoter, NaCl concentration of 150–400 mM and reported higher Na^+^ concentrations in leaves of overexpressing plants under salt treatment, while K^+^ changes were not consistent between the species (Gao et al., 2006; Zhao et al., 2006; Bao et al., 2009; Bhaskaran and Savithramma, 2011; Li et al., 2014). Gene of vacuolar H^+^-pyrophosphatase was among salinity tolerance determinants in barley (Shavrukov et al., 2013). Overexpression of vacuolar H^+^-PPase had also an effect on the whole physiology of plants, for example, increasing root growth via probably auxin transport-associated genes, antioxidant enzymes activities and photosynthetic rate in tobacco (Li et al., 2014). Without salt stress the transgenic plants overexpressing H^+^-pyrophosphatase under control of *35S* promoter demonstrated phenotypes either similar to non-transformed plants (Zhao et al., 2006; Bao et al., 2009; Bhaskaran and Savithramma, 2011), or exhibited improved morphological features (Kumar et al., 2014; Li et al., 2014) or lower osmotic potential in leaves (Gao et al., 2006). Salt treatment for non-transgenic plants resulted in both up- and down-regulation of vacuolar H^+^-PPase in different species, therefore suggesting an important role of vacuolar H^+^-ATPase in responses to the stress factor (reviewed in: Silva and Gerós, 2009).

Another candidates for overexpression are vacuolar *NHX* genes. Overexpression of *AtNHX1* increased salinity tolerance in *Arabidopsis* to 200 mM NaCl, the overexpressing plants accumulated more Na^+^ compared to wild type and demonstrated higher Na^+^/H^+^ exchange activity in isolated leaf vacuoles (Apse et al., 1999). The approach of overexpressing *AtNHX1* to improve salinity tolerance proved to be successful for tomato; the transgenic plants accumulated more sodium in leaves but not in fruits at 200 mM NaCl (Zhang and Blumwald, 2001). Cotton plants with *AtNHX1* from *Arabidopsis* (He et al., 2005), rice overexpressing *SsNHX1* from halophyte *Suaeda salsa* (Zhao et al., 2006), tomato with heterologous NHX from *Pennisetum glaucum* (Bhaskaran and Savithramma, 2011) also showed increased salinity tolerance. Overexpression of *NHX* did not influence the phenotype of plants under control conditions (Apse et al., 1999; Zhang and Blumwald, 2001; He et al., 2005; Zhao et al., 2006; Bhaskaran and Savithramma, 2011).The results with heterologous expression or overexpression of NHX transporters lead to conclusions that the gene is among determinants and potential candidates for engineering salinity tolerance (e.g., Rodríguez-Rosales et al., 2009; Peleg et al., 2011 with more references for successful overexpression of *NHX* to increase salinity tolerance in sugar beet, wheat, maize and the other plants). However, the overexpression of *NHX* was not tissue-specific and under the control of strong promoters, one report did not confirm increase in salinity tolerance in *Arabidopsis* overexpressing *AtNHX1* (Yang et al., 2009). Expression in a tissue-specific manner could be the next step for using NHX to increase salinity tolerance.

The amazing simplicity of the idea to play with the expression of known and functionally well characterized transporters and get salt tolerant or salt sensitive plants is applied to plasma membrane SOS1 Na^+^/H^+^ antiporters and Na^+^ or Na^+^/K^+^ HKT transporters. SOS1 is expressed in (1) epidermal root cells where it participates in sodium eﬄux and in (2) xylem parenchyma cells where SOS1 may load Na^+^ to xylem under moderate salinity and unloads Na^+^ under high salinity or has more complex mode of xylem loading/unloading (Shi et al., 2000, 2002; Pardo et al., 2006; Oh et al., 2007, 2009a; Olías et al., 2009). *Arabidopsis* mutants with defects in gene of SOS1 exhibited strong growth inhibition under salt treatment (Wu et al., 1996), which was rescued in *sos1* mutant by overexpression of *SOS1* gene under *35S* promoter (Shi et al., 2000). Overexpression of *SOS1* gene in wild type plants under *35S* promoter enhanced salinity tolerance of *Arabidopsis* at 100–200 mM NaCl (Shi et al., 2003; Yang et al., 2009), reduced sodium accumulation in shoots and sodium concentration in xylem sap (Shi et al., 2003). Further on overexpression of *SOS1* from *A. thaliana* increased salinity tolerance in transgenic tobacco (Yue et al., 2012) and in transgenic tall fescue (Ma et al., 2014). *SOS1* gene from durum wheat conferred salinity tolerance to *sos1* mutant of *Arabidopsis* (Feki et al., 2014). Interestingly, the effects of overexpression were observed under salt treatment, while in the absence of stress no differences were observed in growth or morphology between wild-type plants and the transgenic lines. Disruption of SOS1 activity by RNA interference in *Thellungiella* on the opposite resulted in the loss of tolerance of the halophyte indicating importance of Na^+^ eﬄux and essential role of SOS1 in salinity tolerance (Oh et al., 2009a). RNA interference of *SOS1* significantly changed the whole transcriptome of *Thellungiella* (Oh et al., 2007) and vacuolar pH under salt treatment (Oh et al., 2009b) proving the complex nature of metabolic and regulatory networks in plants (**Figures 2** and **6**) and yet the probabilistic chances of success in strict overexpression of specific transporters for salinity tolerance improvement. A more complicated situation emerges due to tissue-specific expression. SOS1 is important for long-distance ion transport and xylem loading/unloading in *Arabidopsis* (Shi et al., 2002; discussed in: de Boer and Volkov, 2003), sodium partioning between plant organs in tomato (Olías et al., 2009) and ion fluxes in root meristem zone (Guo et al., 2009), therefore attempts to express it in specific tissues could increase salinity tolerance to a higher extent.

Genetic modification of salinity tolerance using HKT transporters was also successful. Analysis of *Arabidopsis* plants with mutated *HKT* gene revealed higher salt sensitivity of the mutants under long term stress, higher sodium accumulation in their shoots under mild salinity treatment (Mäser et al., 2002) and suggested that HKT is involved in recirculation of sodium within plants (Berthomieu et al., 2003). Further study confirmed increased sodium in the shoots of *Arabidopsis hkt1;1* mutants and clarified that HKT is important for root accumulation of Na^+^ and Na^+^ uptake from xylem in *Arabidopsis* (Davenport et al., 2007). The next step was to create plants overexpressing *HKT* (Møller et al., 2009). *Arabidopsis* plants overexpressing *AtHKT* under the control of *35S* promoter were compared with plants specifically overexpressing *HKT* in cells of root stele. *Pro35S:HKT1;1* plants were salt sensitive probably due to higher Na^+^ uptake by roots while tissue specific overexpression of *HKT* in stele increased salinity tolerance and reduced sodium accumulation in shoots (Møller et al., 2009). The approach was applied to rice where gene from *Arabidopsis AtHKT1;1* was heterologously expressed in root cortex. It resulted in lower shoot Na^+^ concentrations, improved salinity tolerance and involved up- and down-regulation of several membrane transport genes including vacuolar H^+^-pyrophosphatases (Plett et al., 2010). Overexpression of HKT had none (Møller et al., 2009; Plett et al., 2010; Mian et al., 2011) or slight inhibiting pleiotropic effect on growth without NaCl depending on type of promotor for expression and on plant line studied (Møller et al., 2009; Plett et al., 2010). HKT transporters proved to be important for Na^+^ exclusion in wheat and were transferred from durum wheat to bread wheat by interspecific crossing; the genes gave beneficial effects including higher K^+^/Na^+^ ratio in leaves under saline conditions (James et al., 2011). Some plants including barley accumulate Na^+^ in shoots; overexpression of barley *HvHKT2;1* under *35S* promoter in barley increased salinity tolerance at 100 mM NaCl, but opposite to *Arabidopsis* increased Na^+^ concentration in xylem and Na^+^ accumulation in barley leaves (Mian et al., 2011). Taken together the results set HKT transporters to potential candidates for engineering salinity tolerance and among the determinants of the trait (reviewed in: Horie et al., 2009; Almeida et al., 2013; Maathuis et al., 2014) together with the above mentioned NHX1, SOS1 and presumably new studied transporters, e.g., similar to CHX21 from *Arabidopsis* (Hall et al., 2006).

Genes which are important for salinity tolerance in the other groups of organisms and not present in higher plants could also be potential candidates for engineering the trait. Sodium pumping ATPase from moss *Physcomitrella patens* was cloned and expressed under *35S* promoter in rice; plasma membrane expression resulted in higher biomass of transgenic plants compared to control ones after 2 weeks of 50 mM salt treatment. Surprisingly, expression of Na^+^-ATPase did not influence Na^+^ and K^+^ concentrations in transgenic compared to control plants under any tested conditions, so needs more investigation (Jacobs et al., 2011). Recently a new bacterial rhodopsin from *Krokinobacter eikastus* was discovered; it is the first light-driven Na^+^ pump. This rhodopsin was crystallized and resolved using X-ray; the structural basis for Na^+^ transport was revealed (Gushchin et al., 2015; Kato et al., 2015). The photo switchable sodium pump seems a good simple molecular tool. The initial results are already promising and involve the diverse pool of Na^+^-ATPases and transporters from yeast, algae and microbes for improving salinity tolerance in plants.

## Perspectives Of Protein Engineering. Structure-Function Studies And Potential Future For Expression Of Novel Ion Channels, Pumps, And Transporters

Novel opportunities for increasing salinity tolerance in plants are arising with the development of new methods of molecular biology, understanding regulation networks from synthetic biology and growing knowledge about single point mutations changing specific amino acids within molecules of an ion channel or a transporter.

Single amino acid substitutions, e.g., within K^+^ selectivity filter GYG of potassium channels, may change selectivity of the ion channels rendering them from K^+^ selective to non-selective ones (e.g., Heginbotham et al., 1992). Amino acid substitutions within a presumed pore region of HKT transporters are able to alter them from Na^+^ selective to Na^+^ and K^+^ permeable (e.g., Mäser et al., 2002; de Almeida, 2014). Moreover, the single point mutations could be determining for salinity tolerance, e.g., amino acid substitution V395L in rice transporter OsHKT1;5 presumably explained the salt sensitivity of the rice cultivar (Cotsaftis et al., 2012). Specific single point substitution in *Arabidopsis* HAK5 transporter (F130S) over 100 times increased affinity for K^+^ under heterologous expression and reduced inhibition constants for Na^+^ and Cs^+^ (Alemán et al., 2014). Effects of single point mutations on the whole pattern of physiology and on phenotype are well known and better studied in human biomedical science when inherited diseases cystic fibrosis and sickle cell anemia are caused by amino acid substitutions in transport protein CFTR and in hemoglobin, correspondingly.

The knowledge about structure-function correlations of proteins allows to modify the selectivity and create the required properties of ion transport proteins. The way in the direction is to employ the existing and growing information about structure-function of different ion channels and transporters. Next step is to change their ion selectivity and gating properties according to the requirements using single amino acids mutations and to transform the plants of interest in a tissue-specific or cell-specific manner. Examples of tissue-specific transformation already exist (e.g., Møller et al., 2009; Plett et al., 2010) while the new methods and opportunities are progressing enormously (e.g., Brand et al., 2006; Oszvald et al., 2008 etc.). Recent opportunities to directly edit genome using CRISPR–Cas system could overcome some difficulties and directly modify the expressed genes of ion transport proteins (reviewed in e.g., Shan et al., 2013; Kumar and Jain, 2015).

**FIGURE 11:**
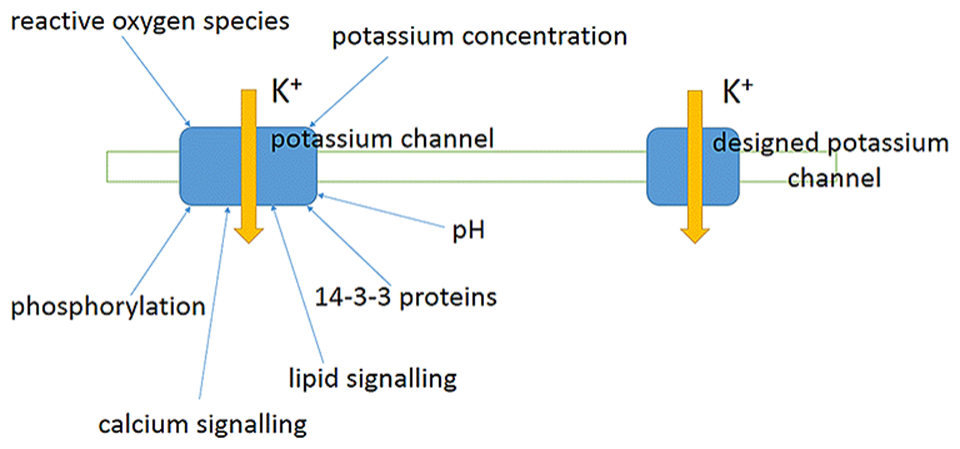
**Novel artificially designed ion channels and ion transporters potentially provide an opportunity to avoid the present evolved protein regulation networks and may be useful for altering ion concentrations, membrane potential and signaling without essentially interacting with the fine-tuning of the existing regulatory networks.** A schematic generalized plant potassium channel in a plasma membrane with several potentially known regulation factors and a model of artificially designed ion channel lacking the evolved known regulation feedbacks.

An alternative approach from synthetic biology is not to modify the existing membrane transport proteins, but to create new ones with desired properties for the further cell-specific transformation (**Figure 11**). The idea is different from what could be assumed at a first glance. Existing biological organisms emerged over the process of long evolution, when previous “building blocks” and elements were used for the future development and often could not be essentially modified due to intrinsic links within organisms and biological systems. It leaves out the question of ideal design, which is mostly not present in biological organisms. Indeed, they are largely predetermined by the previous evolutional history with intrinsic evolutionary trajectories and evolved under multifactor environment (composition of atmosphere, illumination, temperature, salinity and mineral nutrients, water availability etc., while interactions with the other organisms and biotic interactions are often the most important). A simple example is related to temperature. Ion channels in homoeothermic animals like mammals or birds evolved over hundreds of millions of years under stable conditions and hence differ in many properties from ion channels in plants. Sodium and calcium selective ion channels had not been found in plants while in animals they ensure action potentials in neurons and cardiomyocytes. Specialized highly temperature-sensitive ion channel in animals provide temperature sensation (for example transient receptor potential channels, e.g., Ramsey et al., 2006; Myers et al., 2009). Plants are different, they rely on calcium signaling via non-selective cation channels, may have distinct groups of transporters with specific properties, have no known sodium-selective ion channels and use ion channels with low temperature sensitivity (e.g., plant potassium channels and their regulation are reviewed in: Dreyer and Uozumi, 2011; more general review: Hedrich, 2012). Obviously, cell signaling and regulation in plants have numerous specific peculiarities compared to homoeothermic animals. The differences could be reflected in protein structures, therefore simple comparisons may be misleading. Moreover, plant ion channels and transporters are often functioning at membrane voltages below -200 mV while animal membranes are usually not experiencing voltages below -100 mV. Structure and functioning of ion transport proteins from yeast, algae, Protista, and microbes could provide more insights for synthetic proteins for plants.

From the point of the above mentioned it seems that attempts to design novel artificial ion channels and transporters with known characterized ion selectivity filters and voltage sensors adding or excluding specific interacting regulatory elements for the proteins might be productive. The ion channels and transporters when expressed in cell-specific manner under controlled conditions and in defined numbers may avoid fine tuning of regulation and potentially could provide shortcuts in natural signaling networks. The appearing opportunities offer new chances to design salt tolerant plants with previously unknown features and wider ranges of regulation circuits and networks. The potential strategy to increase salinity tolerance includes (1) choice of plant, (2) understanding ion transport and features of salinity tolerance for the plant, (3) determining ion fluxes and ion conductances important for Na^+^ and K^+^ accumulation and compartmentation, (4) modeling ion fluxes and adding/removing in the models ion fluxes and conductances to ensure better nutrient supply and rise of salinity tolerance under salt stress, (5) correlating required changes in ion transport with potential membrane ion transport proteins to realize the ion fluxes, (6) designing specific membrane proteins, (7) expressing the membrane proteins in a tissue-specific manner, (8) checking the salinity tolerance of transgenic plants in laboratory and field experiments. The proposed sequence of events is hypothetical so far and had not been realized yet. However, progress in molecular biology and new ways of thinking from synthetic biology may bring it to fruition and provide new discoveries on the path.

Design of transmembrane proteins is at the very beginning nowadays. Certain structural blocks of the proteins are known better and used for several applications. Voltage-sensors are intensively studied for voltage-gated ion channels and for transporters (Bezanilla, 2008). Moreover, optogenetics needs and already efficiently applies voltage-sensing structural blocks of proteins (Mutoh et al., 2012; Cao et al., 2013; Zou et al., 2014). Selectivity filters of ion channels and transporters are also known and under investigation. Addition of regulatory domains for binding depends on our current information and new endeavors to understand protein–protein interactions and regulation. The further tissue-specific expression and cell-specific localisation of the proteins to be realized via signal peptides and tissue-specific promoters. Problems, strategies and perspectives for engineering novel membrane proteins and re-engineering the existing ones are widely discussed and reviewed (e.g., Grosse et al., 2011; Subramanyam and Colecraft, 2015). *De novo* design, synthesis confirmed by X-ray structure of Zn^2+^-transporting four-helix transmembrane protein bundle is a recent experimental advance (Joh et al., 2014). The Zn^2+^-transport activity of the novel protein was confirmed in functional assays (Joh et al., 2014) proving feasibility of the approach and setting engineering of membrane proteins to a new higher level.

## Conclusion and Perspectives

Development of agriculture often coincides with salinization of the used land, which is later not available for agricultural plants and inhabited by natural halophyte plants. The problem exists since collapse of Sumer civilization about 4000 years ago due to improper agricultural techniques. Recent decades added modern plant physiology, biophysics, molecular and systems biology to pure agriculture and breeding for creating salt-tolerant crops and agriculturally important plants. New information about individual ion transport systems gave solid physical basis for improving salinity tolerance of plants. Emerging opportunities to overexpress individual genes allowed to sharply increase salinity tolerance in laboratory trials. Growing knowledge in protein engineering and synthetic biology sets novel aims and horizons for producing artificial proteins with predefined transport properties and for designing new regulation networks. Potentially the progress in the direction may lead to partially artificial plants with desired salinity tolerance. The next step would be to fill the huge gap between rapid success in laboratory experiments and field practice. Expectedly the future advances will help to release the problem of salinization of agricultural lands.

## Conflict of Interest Statement

The author declares that the research was conducted in the absence of any commercial or financial relationships that could be construed as a potential conflict of interest.
